# Configurational Entropy Driven High‐Pressure Behaviour of a Flexible Metal–Organic Framework (MOF)

**DOI:** 10.1002/anie.202011004

**Published:** 2020-11-12

**Authors:** Pia Vervoorts, Julian Keupp, Andreas Schneemann, Claire L. Hobday, Dominik Daisenberger, Roland A. Fischer, Rochus Schmid, Gregor Kieslich

**Affiliations:** ^1^ Department of Chemistry Technical University of Munich Lichtenbergstr. 4 85748 Garching Germany; ^2^ Computational Materials Chemistry Ruhr University Bochum Universitätsstrasse 150 44801 Bochum Germany; ^3^ Inorganic Chemistry I Technical University Dresden Bergstr. 66 01069 Dresden Germany; ^4^ Centre for Science at Extreme Conditions and EaStCHEM School of Chemistry The University of Edinburgh Kings' Buildings West Mains Road Edinburgh EH9 3FD UK; ^5^ Diamond Light Source Harwell Science and Innovation Campus Didcot OX11 ODE Oxfordshire UK

**Keywords:** metal–organic frameworks (MOFs), high-pressure properties, molecular dynamics, vibrational and configurational entropy

## Abstract

Flexible metal–organic frameworks (MOFs) show large structural flexibility as a function of temperature or (gas)pressure variation, a fascinating property of high technological and scientific relevance. The targeted design of flexible MOFs demands control over the macroscopic thermodynamics as determined by microscopic chemical interactions and remains an open challenge. Herein we apply high‐pressure powder X‐ray diffraction and molecular dynamics simulations to gain insight into the microscopic chemical factors that determine the high‐pressure macroscopic thermodynamics of two flexible pillared‐layer MOFs. For the first time we identify configurational entropy that originates from side‐chain modifications of the linker as the key factor determining the thermodynamics in a flexible MOF. The study shows that configurational entropy is an important yet largely overlooked parameter, providing an intriguing perspective of how to chemically access the underlying free energy landscape in MOFs.

## Graphical abstract

By combining high‐pressure powder X‐ray diffraction and molecular dynamics simulation we gain insight into the microscopic chemical factors that determine the high‐pressure macroscopic thermodynamics of two flexible pillared‐layer MOFs, identifying configurational entropy as originating from side‐chain modifications as the determining factor of the macroscopic thermodynamics.

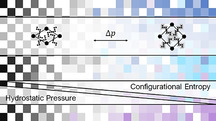

## Introduction

At the centre of applied inorganic chemistry and materials science is the development of structure‐property relationships and the search for materials with (tailored) physicochemical properties of scientific and technological relevance. In this context metal‐organic frameworks (MOFs) have proved as a tantalizing material platform, providing a nearly unlimited parameter space for exploring a wide range of properties such as tuneable nonlinear optical properties,^[1]^ interesting adsorption and desorption behaviour^[2]^ and the use of their pore space for the confined growth of atomically defined inorganic metal halides^[3]^ to name just a few. Intriguingly, the chemical variability of MOFs and coordination networks more generally allows for studying the underlying free energy landscape as a function of small chemical changes, paving the ground for a fundamentally motivated approach for materials design with (smart) dynamic responses to external stimuli.[^4^, ^5^]

A subclass of MOFs, so‐called flexible MOFs, shows large structural flexibility with volume changes exceeding Δ*V*=20 % as response to temperature and pressure variation, and guest adsorption.[^5^, ^6^] Intense research efforts have shown that macroscopic parameters such as topology,[^7^, ^8^] dispersion interactions and vibrational entropy[^9^, ^10^, ^11^, ^12^] as determined by microscopic chemical interactions all contribute to structural flexibility; however, the targeted synthesis of flexible MOFs which concerns the manipulation of macroscopic thermodynamics via chemical changes on a microscopic level is still beyond our knowledge. Therefore, it is not surprising that the number of flexible MOFs[^13^, ^14^, ^15^, ^16^] is still small when compared to the total number of existing MOFs,^[17]^ with MOFs such as ZIF‐4(Zn) (zeolitic imidazolate framework, Zn(im)_2_, with im^−^=imidazolate)[^18^, ^19^, ^20^] and M(bdp) (M^2+^=Fe^2+^ or Co^2+^, bdp^2−^ = 1,4‐benzenedipyrazolate)[^21^, ^22^] being two of several important examples that show large structure flexibility as a function of varying temperature and (gas) pressure.

More generally, flexible MOFs offer the opportunity for the creation of smart materials with distinct responsiveness towards different external stimuli. In this context hydrostatic pressure as external stimulus is gaining more and more attention,[^23^, ^24^] providing fundamental insight into the microscopic chemical interactions as reflected in the macroscopic mechanical properties. Furthermore, studies on the high‐pressure structural behaviour address application‐oriented aspects of MOFs such as stability concerns during material shaping, extrusion and pellet formation for catalytic processes[^25^, ^26^] and their potential as shock absorbers and dampers.[^27^, ^28^, ^29^, ^30^] A few well‐studied examples are MIL‐53 and MIL‐47 (MIL=Matériaux de l'Institut Lavoisier; MIL‐47: V(O)(bdc); MIL‐53: M(OH)(bdc); M^3+^=Al^3+^, Fe^3+^, Cr^3+^, bdc^2−^=1,4‐benzenedicarboxylate),[^31^, ^32^, ^33^, ^34^] which are based on a winerack‐type structure motif. When applying hydrostatic pressure to MIL‐53 and MIL‐47, both materials show a large pore (**lp**) to narrow pore (**np**) phase transition. Importantly, tunability of the transition pressure was recently shown by Yot et al., who implemented functional groups (‐Br and ‐CF_3_) into the MIL‐47(V) framework,^[28]^ thereby accessing the phase transition thermodynamics. In contrast to flexible MOFs it is worth mentioning that non‐flexible MOFs tend to amorphise at relatively low hydrostatic pressures (*p*<0.1 GPa),[^35^, ^36^] emphasising the relatively low bonding energy to volume ratio and drawing a clear line between flexible MOFs and their rigid counterparts. Thus, it is evident that studying the structural response of MOFs to hydrostatic pressure is fundamentally insightful and technologically highly relevant.

**Figure 1:**
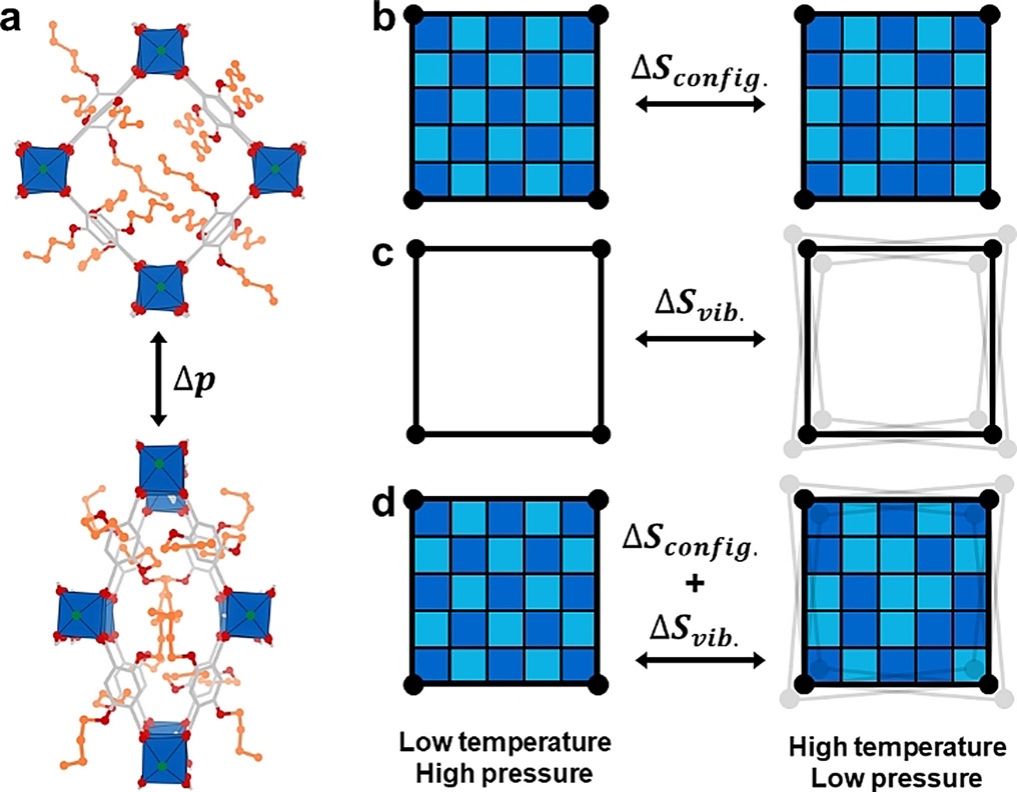
a) shows the **lp** to **np** phase transition of Cu_2_(DB‐bdc)_2_dabco as a function of increasing pressure as extracted from simulations. The view of the structures is along the axis of the dabco pillars; blue polyhedrons represent the coordination sphere of the Cu centres; C grey, O red, N green, DB side chains orange. H atoms are omitted for clarity. b)–d) schematically summarise the different situations of how entropic effects can govern the macroscopic physicochemical properties as a function of temperature and pressure. Most flexible MOFs are best described by situation (c) in which the vibrational entropy dominates, whilst the thermodynamics in Cu_2_(DB‐bdc)_2_dabco exhibit large contributions from configurational entropy (b) and is best described by (d). Light and dark blue squares represent the spatial degrees of freedom (of the side chains) which are higher in the case of the **lp** (high T, low p) phase.

Whilst recognizing the increasing number of high‐pressure studies that have appeared in recent years, it can be observed that the overall number of such studies is still limited, arguably due to experimental limitations.[^37^, ^38^, ^39^, ^40^] One class of flexible MOFs of which the high‐pressure properties still remain entirely unknown is the series of pillared‐layer MOFs with the general formula M_2_(fu‐bdc)_2_dabco (M^2+^=Cu^2+^, Co^2+^, Ni^2+^, Zn^2+^; fu‐bdc^2−^=2,5‐functionalised‐bdc; dabco=1,4‐diazabicyclo[2.2.2]octane).[^41^, ^42^] Here we use the abbreviation fu‐MOFs, emphasising the possibility of linker functionalisation (fu) in this MOF series. In general, fu‐MOFs are built from paddlewheel metal nodes which together with linear bdc^2−^ linkers form 2D sheets with **sql** topology, see Figure 1 a. These sheets are pillared by dabco molecules to build a 3D network with **pcu** topology. It has been shown that the temperature and gas sorption behaviour of fu‐MOFs can be controlled via functionalisation of the bdc^2−^ linker which can render the MOFs flexible.[^43^, ^44^, ^45^, ^46^] For instance, Zn_2_(BME‐bdc)_2_dabco (BME=bis(methoxyethoxy)) contracts to a **np** phase after guest removal and shows a **np** to **lp** phase transition at *T*=493 K.^[47]^ In other words, side chain modifications alter the underlying free energy landscape as determined by dispersion interactions and contributions from vibrational and configurational entropy (*cf*. Figure 1 b–d), emphasising the delicate thermodynamic balance that exists in the M_2_(fu‐bdc)_2_dabco series and in flexible MOFs in general.[^9^, ^11^, ^12^] The response of M_2_(fu‐bdc)_2_dabco to hydrostatic pressure is yet entirely unexplored, providing us with another fascinating angle from which to probe the free energy landscape of these materials as a function of chemical changes.

In this work we explore the high‐pressure response and thermodynamics of Cu_2_(DB‐bdc)_2_dabco (DB=2,5‐dibutoxy) and compare it to the properties of the parent material Cu_2_(bdc)_2_dabco by combining state‐of‐the‐art high‐pressure powder X‐ray diffraction (HPPXRD) and atomistic resolved force field molecular dynamics simulations. Often fu‐MOFs undergo a (reversible) **lp** to **np** phase transition upon guest removal from the pores,[^41^, ^42^] but it was previously shown that Cu_2_(DB‐bdc)_2_dabco remains in the **lp** phase,^[48]^ making it an intriguing example to study its high‐pressure behaviour. Experimental HPPXRD provides us with the high‐pressure responsiveness of the materials to hydrostatic stress which is characterised by the bulk modulus (*K*), the transition pressures and the onset of amorphisation processes. The experiments are complemented with molecular dynamics simulations, monitoring the phase transitions and assessing the underlying energetics computationally. The simulations are based on our recently re‐parameterized MOF‐FF force field (MOF‐FF) for the accompanied side chain library,^[49]^ which allows us to explicitly account for side chain dynamics. The combination of experimentation and theory leads to a detailed picture of the underlying thermodynamics where we identify Cu_2_(DB‐bdc)_2_dabco as the first example in which configurational entropy dictates the observed physicochemical properties.

## Results and Discussion

### General approach

We start by analysing the experimental HPPXRD data of Cu_2_(bdc)_2_dabco and Cu_2_(DB‐bdc)_2_dabco. From this data, the presence of phase transitions and amorphisation processes is observed by visual inspection. In a subsequent quantitative analysis, the evolution of the unit cell volume as a function of pressure is extracted from which the bulk moduli are obtained. These experimental data are used for benchmarking the outcomes of the molecular dynamics simulations, guiding us in the analysis and interpretation of the results. The computational approach is based on the protocol introduced by Rogge et al.,^[50]^ computing the *p*(*V*) equation of state and the thermodynamic properties along the volume change from the **lp** to the **np** form. Since we recently observed a strong dependency of the outcome of such simulations on the initial configuration of the alkoxy side chains in materials such as Cu_2_(DB‐bdc)_2_dabco and Zn_2_(DB‐bdc)_2_dabco,^[49]^ we pay particular attention to such potential bias by comparing computational results to experiments. This provides us with what we believe is highest‐accuracy thermodynamic information of the **lp** to **np** phase transition which is only accessible through molecular dynamics simulations due to the large number of structural distortions such as various number of side chain conformations.

### Experimental observations

**Figure 2:**
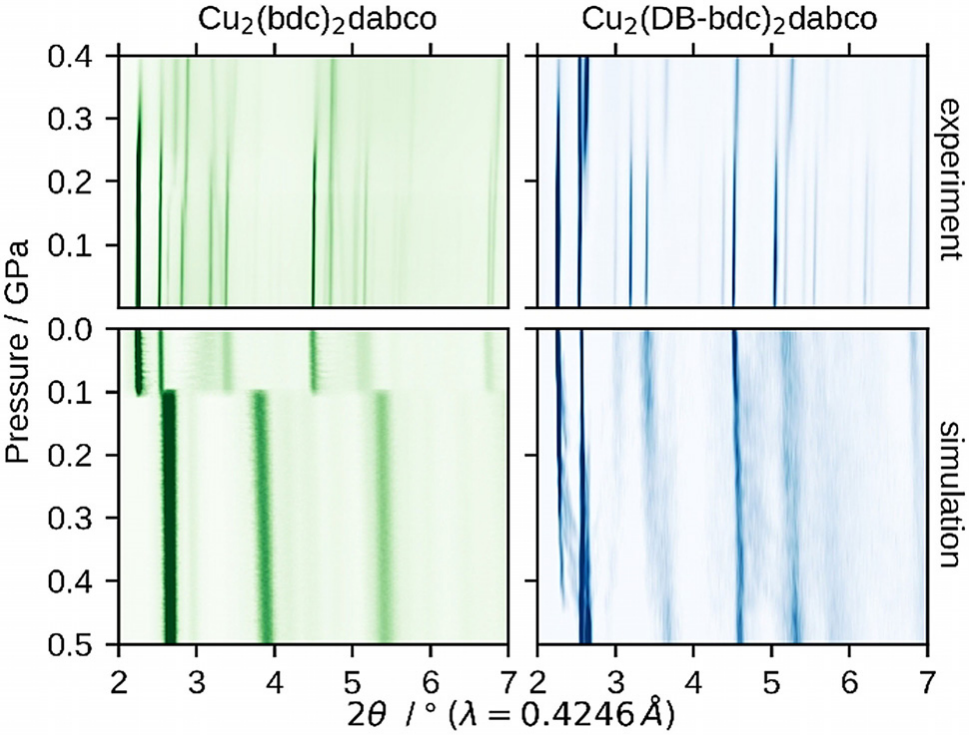
Experimental and simulated HPPXRD patterns. Cu_2_(bdc)_2_dabco (left, green) and Cu_2_(DB‐bdc)_2_dabco (right, blue), as measured (top) and extracted from *NPT* trajectories (bottom). The experimental observations are well reproduced by theory, including the relatively broad range of phase transition for Cu_2_(DB‐bdc)_2_dabco.

The HPPXRD experiments were performed in the pressure range of *p*=ambient−0.4 GPa (Δ*p* per step=0.025–0.05 GPa) on a custom‐built high‐pressure cell^[51]^ operated at the Diamond Light Source beamline I15 with a wavelength of *λ*=0.4246 Å. Contour plots of the HPPXRD data of Cu_2_(bdc)_2_dabco and Cu_2_(DB‐bdc)_2_dabco are shown in Figure 2. A stack plot of all collected HPPXRD patterns is given in the ESI (*cf*. Figure S1 and S2), which includes a complete list of cell parameters, volumes, full width at half maximum (fwhm) and *R*_wp_ values as obtained from a Pawley profile fit analysis.^[52]^ For Cu_2_(bdc)_2_dabco a peak shift to higher 2*θ* angles with increasing pressure resulting from the decreasing unit cell volume is observed, see Figure 2. At approximately *p=*0.175 GPa two reflections of relatively weak intensity jump from 2*θ*=2.63° and 2.81° to 2.75 and 2.89° with increasing intensity relative to the (100) at 2*θ*=2.25° and (001) at 2*θ*=2.53°. Furthermore, a peak broadening and intensity loss of the other reflections occurs, which is ascribed to a combination of a pressure‐induced phase transition and pressure‐induced amorphisation. The analysis of the fwhm of the reflections as a function of pressure shows an increasing fwhm from *p*=0.2 GPa onwards (*cf*. ESI Figure S9). After reaching the maximum pressure (*p*=0.4 GPa), the pressure was released in one step to probe for reversibility. We find partial reversibility as the cell parameters are comparable to those at *p*=0.2 GPa so but with a reduction of the intensity to 1/10 compared to the initial ambient measurement. This provides another indication for the combination of a phase transition and pressure‐induced amorphisation. The results are in general agreement with what has been described recently in a combined computational and Hg intrusion study on Cu_2_(bdc)_2_dabco.^[53]^ In contrast to the reported results, we follow the process in situ via HPPXRD, observing partial reversibility of the phase transition of Cu_2_(bdc)_2_dabco which is not seen in the Hg intrusion experiments.

Looking at the high‐pressure behaviour of Cu_2_(DB‐bdc)_2_dabco, we find a different high‐pressure responsivity. Notably, unlike most other members of the M_2_(fu‐bdc)_2_dabco material series, Cu_2_(DB‐bdc)_2_dabco remains in its **lp** form after guest removal and yet no low temperature **np** phase has been observed.^[48]^ We speculate that the **lp** to **np** phase transition occurs at temperatures below *T*=100 K, not accessible by typical lab X‐ray tools; however, this makes Cu_2_(DB‐bdc)_2_dabco an interesting candidate for a high‐pressure study. Like its parent MOF Cu_2_(bdc)_2_dabco, Cu_2_(DB‐bdc)_2_dabco crystallises in a tetragonal space group, see ESI for stack plots and details of the Pawley profile fits. Up to *p*=0.125 GPa an isotropic compression takes place and starting from *p*=0.15 GPa the phase transition to the **np** form occurs. The phase transition occurs over a broad pressure range and is seen as a displacive phase transition according to Buerger^[54]^ rather than a collapse of the network. In contrast to Cu_2_(bdc)_2_dabco the phase transition is fully reversible and the material switches back to its initial **lp** form with similar cell parameters compared to the initial measurement after pressure release.

The relatively large peak shifts in the contour plots in Figure 2 suggest that both materials are rather soft materials, which can be quantified by the bulk modulus (*K*). The bulk modulus is defined as the inverse of the compressibility and is a measure of mechanical resistance of a material towards volumetric changes under hydrostatic pressures, that is, $K=-V_{0}\partial p/\partial V$ with $V_{0}$ being the volume of the unit cell at ambient pressures and ∂p/∂V the change of pressure as function of volume variation. Taking into account the onset pressure of amorphisation of Cu_2_(bdc)_2_dabco and the phase transition of Cu_2_(DB‐bdc)_2_dabco, only data up to *p*=0.175 GPa and 0.125 GPa, respectively were fitted to a 2^nd^ order Birch‐Murnaghan Equation of State (BM EoS) using EoSFit‐7c^[55]^ to obtain the bulk moduli of the ambient phases (see ESI for fits of the BM EoS and stress‐strain plots). The obtained bulk moduli are *K(*Cu_2_(bdc)_2_dabco)=14.03±0.20 GPa and *K(*Cu_2_(DB‐bdc)_2_dabco)=13.46±0.22 GPa. Putting these bulk moduli into the context of other reported MOFs (*cf*. ESI Table S4) Cu_2_(bdc)_2_dabco and Cu_2_(DB‐bdc)_2_dabco are neither particularly rigid nor soft. Interestingly, previously reported bulk moduli data for Cu_2_(bdc)_2_dabco by Wieme et al.^[53]^ as obtained from Hg intrusion experiments and simulations are higher with 18.8 GPa and 16.4 GPa, whereas the bulk moduli extracted from the force field simulations herein predict a smaller value of about *K*=11.3 GPa for Cu_2_(bdc)_2_dabco and *K*=10.2 GPa for Cu_2_(DB‐bdc)_2_dabco. This underlines the difficulty to obtain reliable bulk moduli for porous and rather soft materials, highlighting the large challenges related to measurement accuracies and material treatment, potential unknown contributions from defects and the difficulties in reproducing these computationally.

### Computation and thermodynamics

Force field based molecular dynamics simulations of Cu_2_(bdc)_2_dabco and the functionalised Cu_2_(DB‐bdc)_2_dabco were performed to obtain an atomistic picture of the ramifications of side chain functionalisation for the underlying free energy landscape. The challenge in the simulations lies in minimising the bias of the initial linker configuration on the computational outcomes within computationally accessible timescales.^[49]^ For instance, within several nanoseconds a linker flip, that is, a 180° rotation of a phenyl moiety is only very rarely observed. The situation is further complicated by the absence of experimental structures from X‐ray diffraction which include spatially resolved side chain configurations. Such defined side chain configurations were so far only observed in the case of pentoxy functionalised Zn_2_(DPe‐bdc)_2_dabco (DPe=dipentoxy).^[44]^ We therefore constructed initial structural models in silico from the **pcu** network shown in Figure S9 using the weaver code^[56]^ followed by a structure optimization step. For Cu_2_(bdc)_2_dabco one single starting structure is used, whereas for Cu_2_(DB‐bdc)_2_dabco 32 individual starting structures with random phenyl orientations, that is, random torsion angles as shown in ESI Figure S9 were generated and optimized. We would like to point out that the large number of possible configurations is the reason why alternative methods to compute the free energy differences between the **np** and **lp** form based on quantum mechanical methods using internal energies plus (quasi)harmonic approximation of vibrational entropy are not readily applicable here.[^10^, ^11^, ^57^] In contrast, by using force field based simulations, it is possible to harness the computational efficiency and accuracy of our recent parameterisation^[49]^ of MOF‐FF for the flexible side chains to perform extensive sampling of different configurations with respect to the phenyl orientation which leads to different relative positions of the oxo moieties as well as side chain dynamics to capture their configurational entropy.

**Figure 3:**
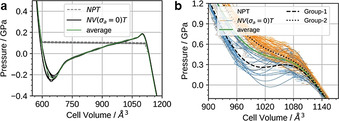
Results of the *NPT* and the *NV*(*σ_a_*=0)*T* simulations. a) Cu_2_(bdc)_2_dabco and b) Cu_2_(DB‐bdc)_2_dabco. *p*(*V*) profiles are obtained from pressure ramp *NPT* simulations averaged with a window of *t*=50 ps (dashed curves) and the respective *p*(*V*) equation of state computed via *NV*(*σ_a_*=0)*T* simulations (thin solid curves) and selected averages (thick curves).

**Figure 4:**
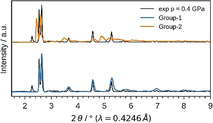
Experimental and simulated PXRD data of Cu_2_(DB‐bdc)_2_dabco at *p*=0.4 GPa. Experimental data are shown in black, the simulations with evidence for structure instability in blue (Group‐1) and with no evidence for structure instability in orange (Group‐2).

After setting up a methodology that allows for evaluating potential bias of the starting configuration on the computational outcomes, *NPT* and subsequent *NV*(*σ_a_*=0)*T* simulations were performed for Cu_2_(bdc)_2_dabco and Cu_2_(DB‐bdc)_2_dabco, see Figure 3, following the recipe described in Ref. [50]. In the pressure ramp *NPT* simulations, which are based on 8 (Cu_2_(bdc)_2_dabco) and 32 (Cu_2_(DB‐bdc)_2_dabco) simulations, evidence for phase transitions can already be observed; however, only from the *NPT* simulations it is not clear whether these simulated transition pressures originate from the underlying physics or from our simulations, and previously, premature phase transition artefacts have been observed.^[50]^ Looking at the *p*(*V*) curves of Cu_2_(bdc)_2_dabco as obtained from *NV*(*σ_a_*=0)*T* calculations the occurrence of a phase transition is confirmed with a transition pressure of *p*=0.2 GPa. In contrast, a slightly different situation is observed for Cu_2_(DB‐bdc)_2_dabco where the transition pressures of each individual *NPT* and *NV*(*σ_a_*=0)*T* simulation are consistent with each other. For the 32 *NV*(*σ_a_*=0)*T* simulations of Cu_2_(DB‐bdc)_2_dabco, the results can be grouped into (*i*) a set of simulations with evidence for a structure instability which we call Group‐1 (Figure 3, blue), and (*ii*) a second set of simulations with the absence of a maximum in the *p*(*V*) curve (Figure 3, orange) here termed Group‐2. From computation alone it is impossible to distinguish between Group‐1 and Group‐2 in terms of relevance and goodness; however, with having the corresponding HPPXRD experiments available, we used a Rietveld method based rating function to compare simulated PXRD patterns from averaging along the *NV*(*σ_a_*=0)*T* trajectories with the experimental PXRD patterns, see ESI for details. Applying this type of rating scheme, we obtain a set of 12 simulations of the initial 32 simulations of which their PXRD patterns are in general agreement with the experiment, see Figure 2, Figure 4 and Figure S16, and Figure S18–S20 for a comparison of lattice parameters. In other words, one subset of starting configurations which includes 12 individual simulations reproduces the experimental observations and will be used from here on for further discussions. For a direct comparison between experimental PXRD pattern and simulated PXRD pattern averaged over Group‐1 and Group‐2, see Figure 4.

A more extensive discussion on the structural differences between the different subsets and their origins based on an analysis of collective variables of the different structural features can be found in the ESI. From analysing the average *p*(*V*) *NV*(*σ_a_*=0)*T* simulations of this subset we obtain a phase transition pressure of *p*=0.29 GPa and by including the variance of the transition pressure within individual simulations, we obtain a window of 0.19 GPa≤*p*_trans_≤0.39 GPa which is in excellent agreement with the experiment.

**Figure 5:**
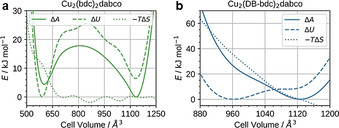
The thermodynamic quantities as obtained from simulation. Helmholtz free energy Δ*A*, internal energy Δ*U* and entropy contribution −*T*Δ*S* as a function of the cell volume *V*. a) Cu_2_(bdc)_2_dabco and b) Cu_2_(DB‐bdc)_2_dabco.

Now we can turn our attention to the details of the underlying thermodynamics as observed from computation. The Helmholtz free energy *A* was computed from *p*(*V*) by numerical integration, and the internal energy *U* obtained from the average of the total energy of a trajectory. For the unfunctionalised material Cu_2_(bdc)_2_dabco (Figure 5 a) we see two distinct minima in Δ*A*, which belong to the ambient and high‐pressure phases. From comparing Δ*A* with Δ*U*, it can be observed that the bistability of Cu_2_(bdc)_2_dabco is mainly driven by Δ*U*, and that the **np** form is destabilised compared to the **lp** form due to its unfavourable −*T*Δ*S*. This behaviour is in agreement with the general rule that the crystallographically less dense structure has a higher vibrational entropy.[^12^, ^58^, ^59^] This tendency has also been observed for MIL‐53(Al)[^11^, ^60^] and MIL‐53(Cr)^[10]^ and has been explained by only considering vibrational entropy contributions. We would like to note that the applied computational approach is not set up to reproduce amorphisation that has been observed in the experiment, that is, the simulations are based on a non‐reactive potential to model the system which restricts any possible bond cleavage; however, we have recently shown that once initiated the **lp** to **np** phase transition of Cu_2_(bdc)_2_dabco occurs rapidly and releases huge amounts of energy as the phase transition wave spreads throughout the crystallite.^[61]^ Approximating the computed work that is released with *W*=*pΔV*=61.6 kJ mol^−1^ per formula unit, and transforming it into kinetic energy of the atoms, the temperature increases by almost *T*=100 K. Therefore, we hypothesise that this energy is so large that it can easily overcome the energy of the coordination bonds in the MOF system, leading to amorphisation.

For the functionalised material Cu_2_(DB‐bdc)_2_dabco we observe a distinctly different behaviour (Figure 5 b). Firstly, the two minima in Δ*U* are less pronounced, broadly reflecting a shallower free energy landscape. The **lp** form of Cu_2_(DB‐bdc)_2_dabco benefits from seemingly large entropic contributions compared to Cu_2_(bdc)_2_dabco with −*T*Δ*S* monotonically increasing as a function of decreasing cell volume that is, increasing pressure. Given that the only difference between Cu_2_(DB‐bdc)_2_dabco and Cu_2_(bdc)_2_dabco is the alkoxy functionalisation of the bdc^2−^ linker, we ascribe the trend in −*T*Δ*S* to configurational entropy originating from side chain functionalisation. Qualitatively, reducing the available pore volume via the application of hydrostatic pressure results in a situation where side chains have a reduced number of spatial orientations, in turn reducing contributions from configurational entropy as a function of decreasing pore volume (increasing pressure). Under the assumption that entropic contributions can be divided into configurational and vibrational, and that the latter can be qualitatively described via a harmonic approximation, we can further quantify the single contributions. We optimized the **lp** and **np** forms of Cu_2_(bdc)_2_dabco and Cu_2_(DB‐bdc)_2_dabco and computed contributions from vibrational entropy at *T*=300 K as obtained within the harmonic approximation using phonopy.^[62]^ For the unfunctionalised MOF Cu_2_(bdc)_2_dabco we obtain −*T*Δ*S*=16.7 kJ mol^−1^, which is in agreement with the entropy penalty shown in Figure 5 a. In contrast, a value of −*T*Δ*S*=21.1 kJ mol^−1^ is obtained for the functionalised material Cu_2_(DB‐bdc)_2_dabco which is significantly lower when compared to the value of approximately −*T*Δ*S*=41.8 kJ mol^−1^ as obtained from *NV*(*σ_a_*=0)*T* simulations. Therefore, contributions from configurational entropy are calculated to −*T*Δ*S*=20.7 kJ mol^−1^, superimposing effects from vibrational entropy and presenting an important factor in driving the entropic penalty of the functionalised MOF as the cell volume decreases. Although we here provide the exact values of vibrational and configurational entropic contributions, we would like to note that these should be regarded as qualitative. Comparing this situation to other flexible MOFs, we here show that Cu_2_(DB‐bdc)_2_dabco represents the first example in which the physicochemical properties of the flexible MOF are governed by configurational entropy. For instance, it is established that the phase transition in ZIF‐4(Zn) is based on a delicate balance between dispersion interactions and vibrational entropy, where the **lp** (high temperature) phase of ZIF‐4(Zn) comes with a gain in vibrational entropy driven by a softening of low frequency modes.^[12]^ Likewise, the **np** to **lp** phase transition in the well‐studied MOF MIL‐53 has been shown to be driven by vibrational entropy only.[^9^, ^10^, ^60^] Therefore, most flexible MOFs are best described by the situation shown in Figure 1 b, whilst Cu_2_(DB‐bdc)_2_dabco is the first example in which both configurational and vibrational entropy with a weighting of approximately 1:1 exists (Figure 1 d). Therefore, Cu_2_(DB‐bdc)_2_dabco is a fascinating example of how small chemical modifications such as alkoxy side chains can be used to introduce configurational entropy, alter the underlying free energy landscape and therewith the macroscopic properties of a MOF. We expect that the introduction of configurational entropy via side chain modification is a general phenomenon, providing another fascinating angle of how to optimize the free energy landscape to render a rigid MOF structurally flexible.

## Conclusion

In conclusion we investigated the pressure dependent responsivity of the prototypical flexible MOFs Cu_2_(bdc)_2_dabco and Cu_2_(DB‐bdc)_2_dabco by combining state‐of‐the‐art experimentation with computation. We observe side chain dependent high‐pressure behaviour, underlining the opportunities that come with side chain functionalisation as a tool to manipulate the physicochemical properties of a MOF. The molecular dynamics simulations provide in‐depth insight into the thermodynamic factors that govern the free energy landscape, once again highlighting the important role of entropic contributions in the large structural flexibility of MOFs. In contrast to other flexible MOFs we identify configurational entropy as governing factor, drawing a clear line between the underlying thermodynamics of Cu_2_(DB‐bdc)_2_dabco and other flexible MOFs. The results suggest the use of configurational entropy as a lever for tuning the underlying free energy landscape, a factor which has been broadly overlooked as design parameter so far.

Our works also prove that the combination of experiment and theory can provide a detailed picture of the factors that link the microscopic arrangement of atoms with macroscopic physicochemical properties in flexible MOFs. Whilst in situ experimental methods such as solid state nuclear magnetic resonance spectroscopy and single crystal X‐ray diffraction during gas sorption experiments have greatly improved over the years,[^63^, ^64^] it should be underlined that solely from experiment it is not possible to draw conclusions on the underlying thermodynamics. This knowledge, however, is crucial for the development of design rules for stimuli responsive materials, guiding experimentalists from trial‐and‐error synthesis to the targeted synthesis of compounds. Looking forward, it seems that the great effort of the community on flexible MOFs has led to a thorough picture on the different microscopic factors that determine the macroscopic properties. Thus, we believe it is now the opportune time that theoreticians and experimentalists with different backgrounds and expertise join forces, even stronger, and to combine existing knowledge for the identification of synthetic principles that foster the discovery of new flexible MOFs.

## Conflict of interest

The authors declare no conflict of interest.

## Supporting information

As a service to our authors and readers, this journal provides supporting information supplied by the authors. Such materials are peer reviewed and may be re‐organized for online delivery, but are not copy‐edited or typeset. Technical support issues arising from supporting information (other than missing files) should be addressed to the authors.

Supplementary
