# Supplementary material for: Configurational Entropy Driven High‐Pressure Behaviour of a Flexible Metal–Organic Framework (MOF)
Source: Angew Chem Int Ed Engl. 2020 Nov 12;60(2):787–93. doi: 10.1002/anie.202011004 (PMC7839482; doi:10.1002/anie.202011004)
Supplement: Supplementary file 1 — Supplementary [file ANIE-60-787-s001.pdf]

Angewandte Chemie
Eine Zeitschrift der Gesellschaft Deutscher Chemiker

Supporting Information

Configurational Entropy Driven High-Pressure Behaviour of a Flexible Metal–Organic Framework (MOF)

Pia Vervoorts+, Julian Keupp+, Andreas Schneemann, Claire L. Hobday, Dominik Daisenberger, Roland A. Fischer, Rochus Schmid, and Gregor Kieslich**

anie_202011004_sm_miscellaneous_information.pdf

SUPPORTING INFORMATION

Content

Methods and Syntheses 2
High-Pressure Powder X-ray Diffraction 3
Pawley Profile Fit Analysis........ 5
Bulk Modulus 13
Bulk Moduli of Various MOFs 14
NiDMG as Reference Material........ 15
Computational Details........ 18
Cell Parameters 21
Thermodynamics of $Cu_2(DB\text{-}bdc)_2dabco$: Internal Energy, Free Energy and Entropy 24
Computing the Work Released during the Phase Transitions 25
Structural Details via Collective Variables 26
Variability of the Linker Orientations of the $Cu_2(DB\text{-}bdc)_2dabco$ Simulations 28
References 29

Methods and Syntheses

General information. All chemicals were purchased from commercial suppliers (ABCR, Acros Organics, Alfa Aesar, Sigma Aldrich, TCI) and used without further purification unless otherwise stated. Solvents used for the syntheses and washing steps were reagent grade or higher.

Synthesis and sample activation. The linker H_2DB-bdc was synthesized according to Ref. [1]: Me_2(OH_2-bdc) (4.42 mmol, 1.0 eq.) and K_2CO_3 (20.1 mmol, 4.5 eq.) were suspended in DMF (35 mL). 1-Bromobutane (9.73 mmol, 2.2 eq.) was added drop wise to the yellow solution and the solution was heated to T = 85 °C overnight. The solvent was removed under reduced pressure and NaOH solution (20.1 mmol, 4.5 eq. in 40 mL H_2O) was added to the brown residue. After t = 4 hours of refluxing, the reaction mixture was cooled to room temperature. HCl (~15 %) was added until a colourless solid precipitated. The colourless precipitate was filtered, washed with H_2O and dried in vacuo at T = 70 °C. ^{1}H NMR (200 MHz, DMSO-d_6) δ = 7.25 (s, 1H), 3.97 (t, J = 6.2 Hz, 2H), 1.78 – 1.55 (m, 2H), 1.56 – 1.29 (m, 2H), 0.90 (t, J = 7.3 Hz, 3H) ppm. ^{13}C NMR (63 MHz, DMSO-d_6) δ = 167.32, 150.53, 120.01, 117.63, 52.64 ppm.

The MOFs were synthesized and activated following this protocol: $Cu(NO_3)_2 \cdot 3\ H_2O$ (0.87 mmol, 1.0 eq.), the respective linker (H_2bdc or H_2DB-bdc, 0.87 mmol, 1.0 eq.) and dabco (0.87 mmol, 1.0 eq.) were dissolved in 20 ml DMF and sonicated for 5 min. The sealed vials were placed in an oven and heated to T = 120 °C for t = 48 h under autogenous pressure. The samples were activated via solvent exchange by replacing the reaction solution with fresh DMF (3 x 25 mL) over the course of three days. Afterwards, DMF was replaced by dichloromethane (DCM, 3 x 25 mL). After the last removal of DCM, the samples were dried in vacuo at T = 100 °C. The activated samples were stored in a glovebox under Ar atmosphere.

NMR. The ^{1}H NMR was measured on a Bruker DPX-200 (200 MHz) and the ^{13}C NMR on a Bruker DPX-250 (63 MHz) at T = 298 K. DMSO-d_6 was used as solvent. ^{13}C NMR spectra were measured with an attached proton test (APT). Chemical shifts δ are given in ppm relatively to TMS and are referenced with MestreNova to DMSO-d_6 as an internal standard.

High-pressure powder X-ray diffraction. High-pressure powder X-ray (HPPXRD) data were collected at the Diamond Light Source (beamline I15) within beamtime EE19187-1 using an X-ray energy of 29.2 keV (λ = 0.4246 Å) and a 2D PerkinElmer area detector for data collection. The applied high-pressure cell is described in detail at https://www.imperial.ac.uk/pressurecell/ and Ref. [2]. The activated and ground sample (either Cu_2(DB-bdc)$_2$dabco or Cu_2(bdc)$_2$dabco) was filled together with a pressure transmitting medium into a PTFE plastic capillary (inside diameter 1.8 mm) and then sealed with Araldyte-2014-1. To ensure the activation of the sample, the preparation was done in a glovebox under Ar atmosphere. Silicone oil AP 100 was chosen as the pressure transmitting medium as there is no evidence found in previous high-pressure studies of porous MOFs that it penetrates the pores. Additionally, it is expected to maintain hydrostatic conditions up to pressures of p = 0.9 GPa. The plastic capillary was loaded into the water filled sample chamber. The pressure on the sample is adjusted by increasing or decreasing the water pressure in the sample chamber using a hydraulic gauge pump system equipped with three pressure transducers to control the applied pressure precisely. HPPXRD is performed through two diamond windows in the sample chamber. In total, 16 (Cu_2(bdc)$_2$dabco) and 19 (Cu_2(DB-bdc)$_2$dabco) HPPXRD pattern were collected between p = ambient and 0.40 GPa including one after releasing the pressure to prove the reversibility. The step size varied between Δp = 0.025 – 0.05 GPa. Pawley profile fits of the data were performed using TOPAS V6.[3] The bulk moduli were derived by fitting a 2nd order Birch-Murnaghan equation of state (BM EoS) extracted data. Standard deviations (σ) of lattice parameters and volumes were obtained during the Pawley profile fits and were included in the fitting process of the $V(p)$ data with the software EoSFIT-7c.[4]

High-Pressure Powder X-ray Diffraction

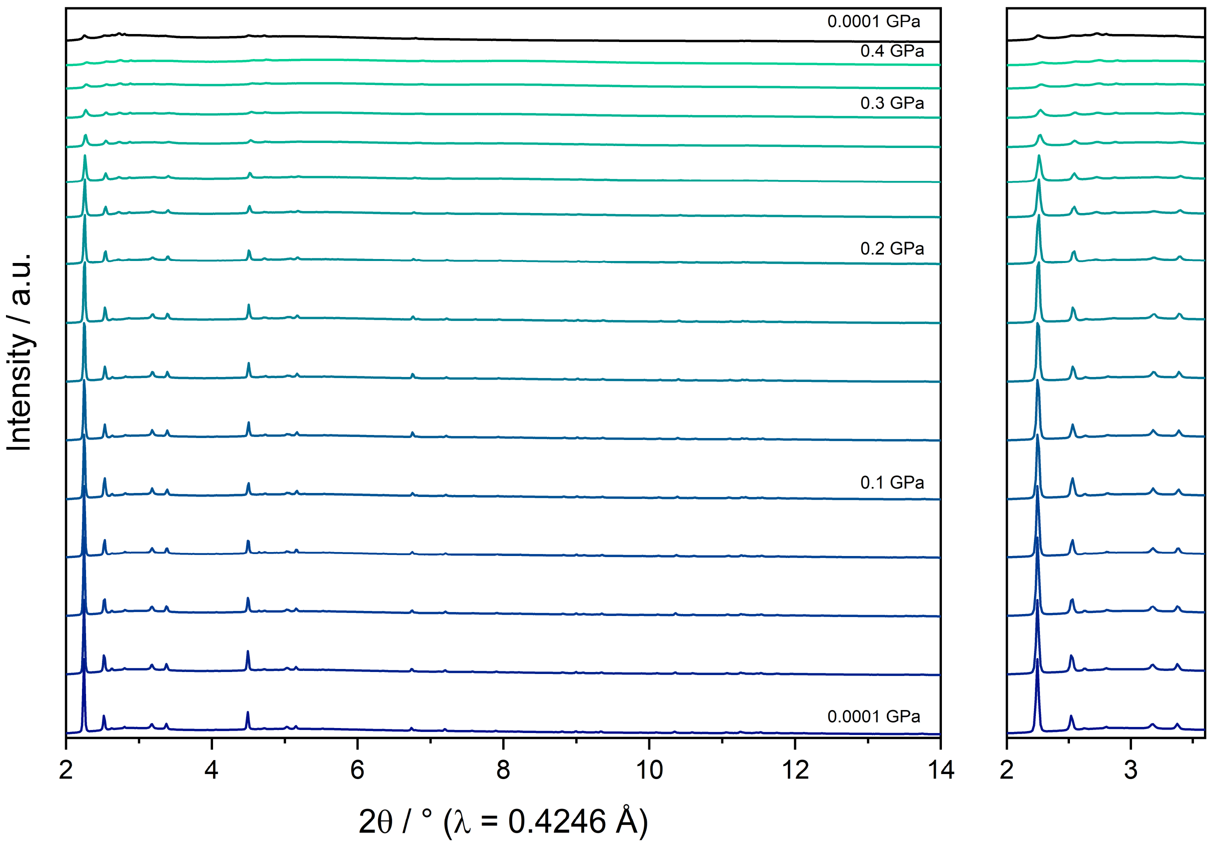

Figure S1. Integrated HPPXRD data of $Cu_2(bdc)_2dabco$ taken at p = 0.0001, 0.025, 0.050, 0.750, 0.100, 0.125, 0.150, 0.175, 0.200, 0.225, 0.250, 0.275, 0.300, 0.350, 0.400 and 0.0001 GPa after the pressure was released (black) and a zoom in between 2θ = 2 and 3.6°.

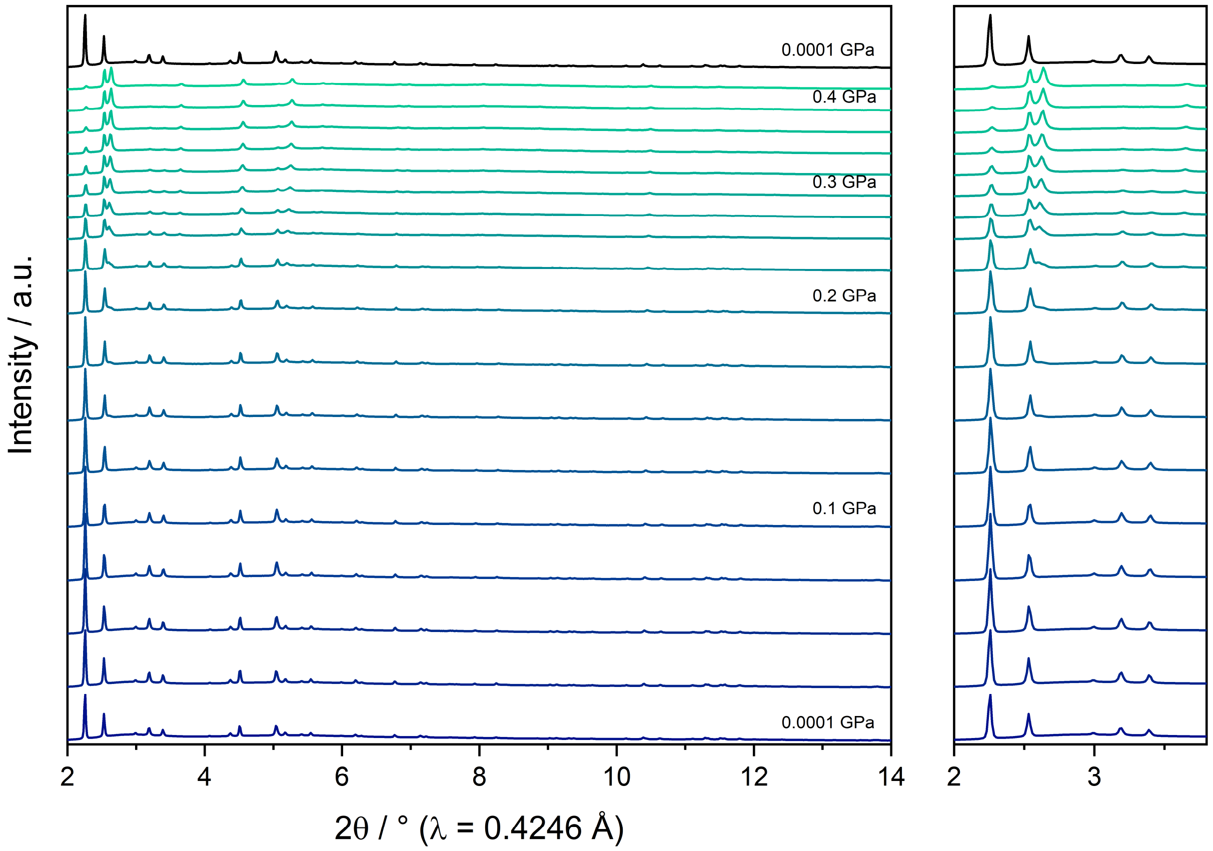

Figure S2. Integrated HPPXRD data of Cu_2(DB-bdc)$_2$dabco taken at p = 0.0001, 0.025, 0.050, 0.075, 0.100, 0.125, 0.150, 0.175, 0.200, 0.225, 0.250, 0.275, 0.300, 0.325, 0.350, 0.375, 0.400, 0.425 and 0.0001 GPa after the pressure was released (black) and zoom in between 2θ = 2 and 3.6°.

Pawley Profile Fit Analysis

Pawley profile fits of the data were performed using TOPAS V6.[3] The standard deviations (σ) of lattice parameters and volumes were obtained and the error of the pressure was estimated as p = ± 0.0020 GPa. Based on the sharp diffraction peaks the presence of diffraction domain sizes in the nanoregime can be excluded.

Cu_2bdc_2dabco was indexed in the space group *P4/mmm* (No. 123) with two minor additional reflections at 2θ = 2.63° and 2.81°, which were observed in previous studies[5] and therefore fitted manually. All cell parameters and their standard deviations are listed in Table S1.

$Cu_2(DB\text{-}bdc)_2dabco$ crystallises like its parent MOF in a tetragonal space group. Although Schwedler *et al.* indexed the Zn analogue in the space group *P4/ncc* (No. 130),[6] the Pawley fits using Topas V6 point out that for the Cu analogue the space group *P4/n* (No. 85) suits best for the **lp** form. The space groups differ in their diffraction conditions (00l : $l = 2n$ for *P4/ncc* but not for *P4/n*)[7] which leads to a doubling of the cell size and reflections that are not observed in the experimental diffraction pattern (*e.g.* 2θ = 3.8, 5.6, 5.9, 6.4° *etc.*). The phase transition comes with a symmetry lowering to the monoclinic space group *P2/c* (No. 13). Similar as reported by Henke *et al.* for the flexible $Zn_2(BME\text{-}bdc)_2dabco$,[8] we observe that the first reflection (corresponding to the (110) reflection) shifts from 2θ = 2.25° to 2θ = 2.65° and the (200) reflection from 2θ = 3.18° to 2θ = 2.63° which is attributed to an elongation of cell parameter *a* and contraction of cell parameter *b*. Henke *et al.* also reported that the (001) reflection at 2θ = 2.53° does not shift, which is different for our material. Here, the (001) reflection is not seen for the np phase because of the reflection conditions of the space group P2/c which are $h0l$: $l = 2n$ and 00l: $l = 2n$.[7] Instead the (010) reflection is seen at 2θ = 2.30°. Note, that it cannot be deduced from the PXRDs as a bulk method, if both phases are observed simultaneously in one particle or exist next to each other in different particles. From p = 0.15 GPa both phases are present and therefore a biphasic fit is performed. All cell parameters and their standard deviations are listed in Table S2 and Table S3.

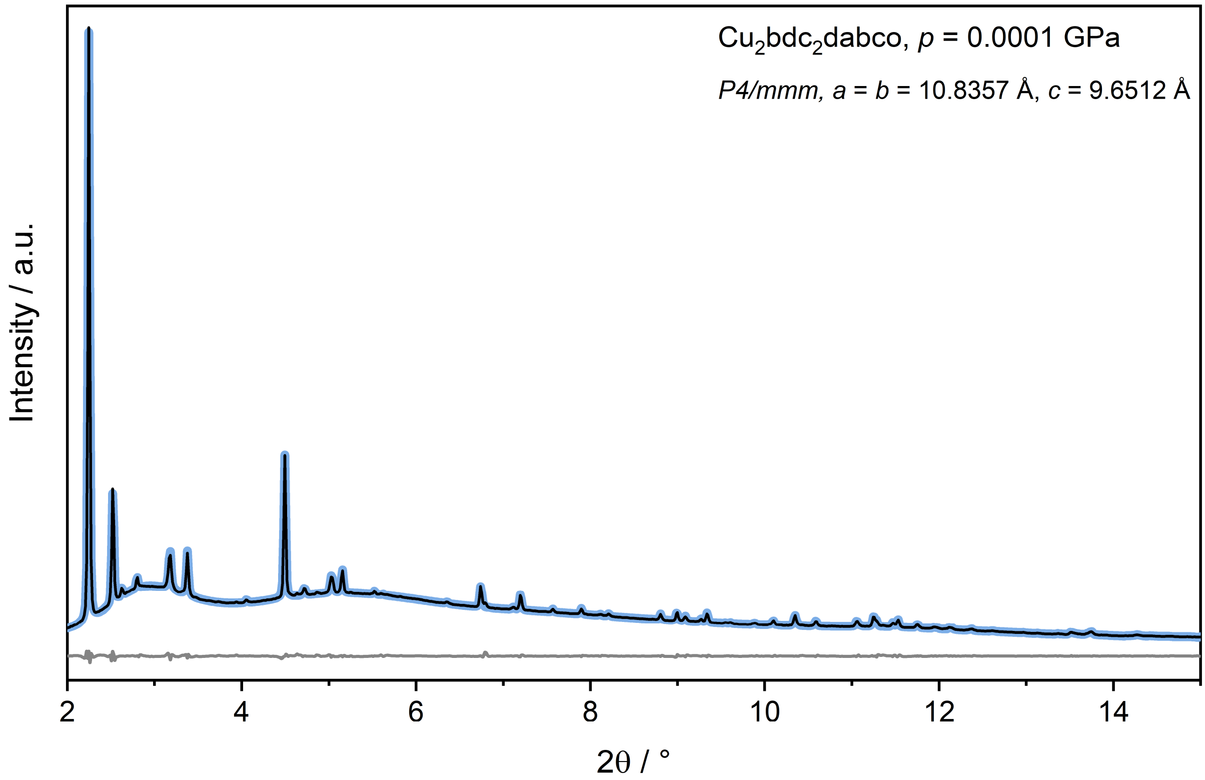

Figure S3. Pawley profile fit for $Cu_2(bcd)_2$dabco at p = 0.0001 GPa. Experimental diffraction pattern in black, Pawley profile fit in blue and difference curve in grey.

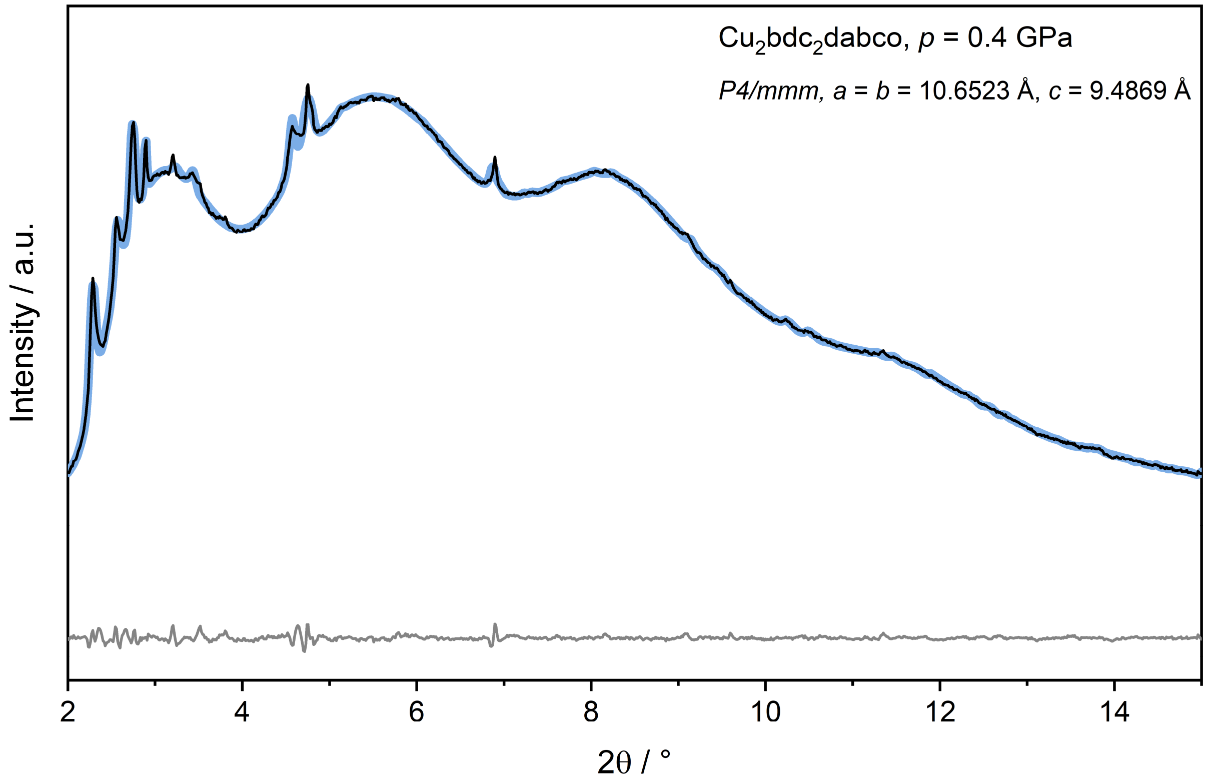

Figure S4. Pawley profile fit $Cu_2(bcd)_2$dabco at p = 0.4 GPa. Experimental diffraction pattern in black, Pawley profile fit in blue and difference curve in grey.

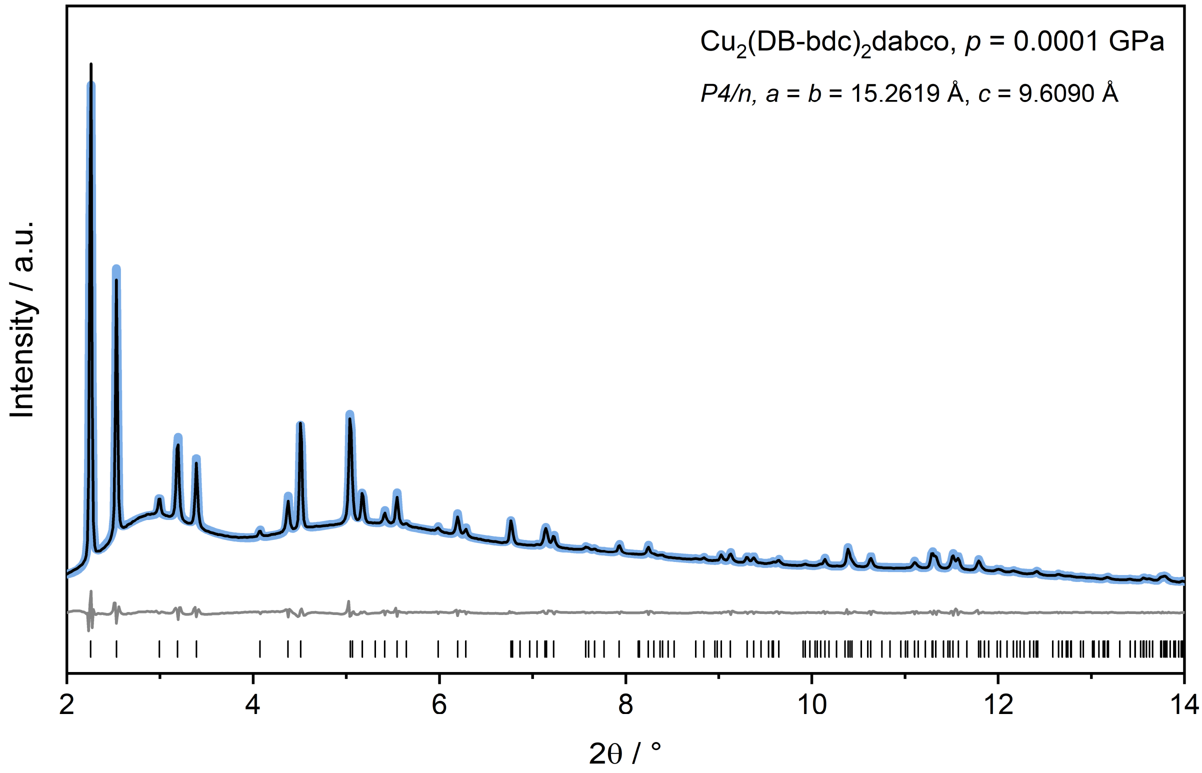

Figure S5. Pawley profile fit $Cu_2(DB\text{-}bcd)_2dabco$ at *p* = 0.0001 GPa. Experimental diffraction pattern in black, Pawley profile fit in blue and difference curve in grey.

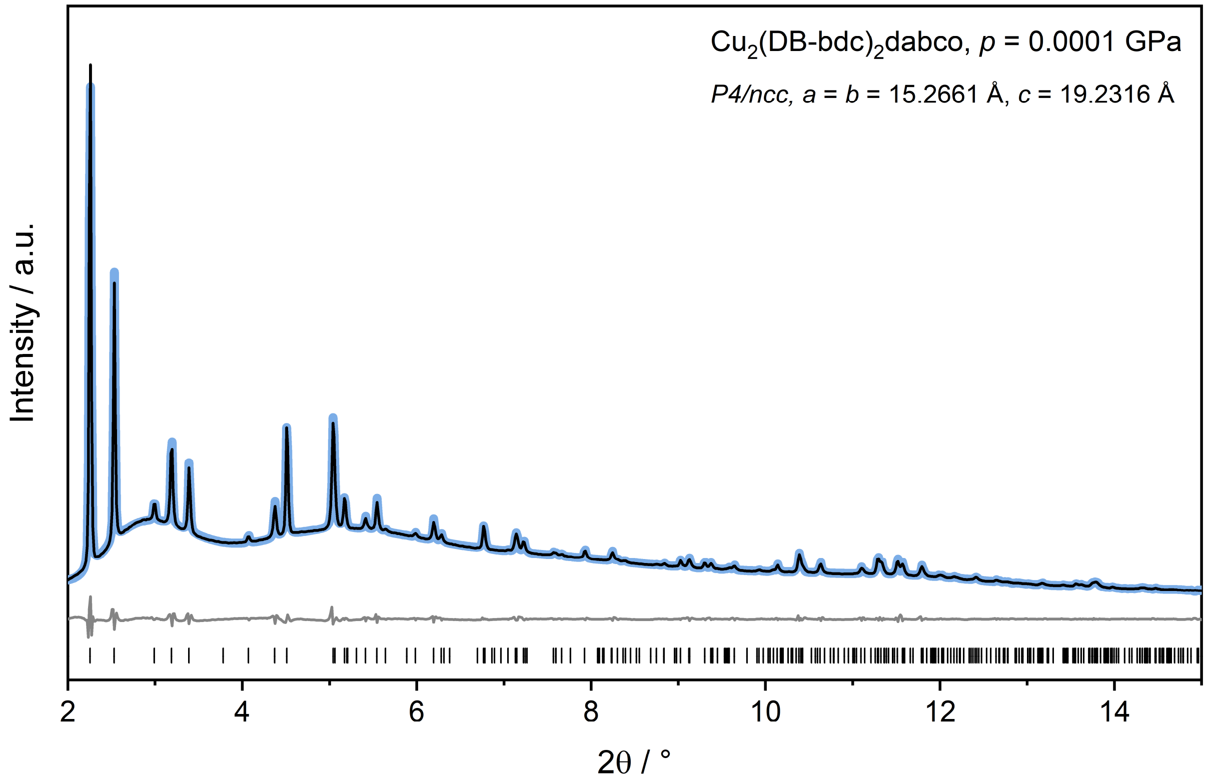

Figure S6. Pawley profile fit $Cu_2(DB\text{-}bcd)_2dabco$ at *p* = 0.0001 GPa with the space group P4/ncc shown as comparison. Experimental diffraction pattern in black, Pawley profile fit in blue and difference curve in grey, position of the reflections shown as black tick marks.

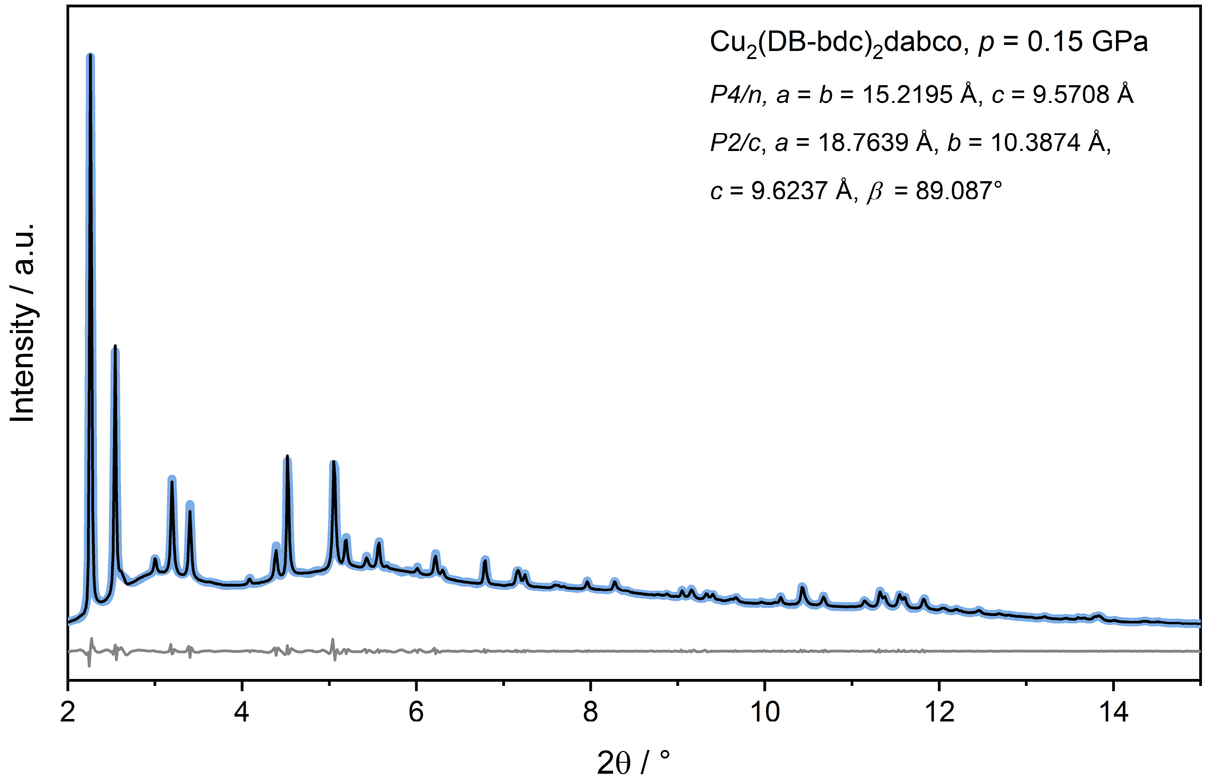

Figure S7. Pawley profile fit $Cu_2(DB\text{-}bcd)_2$dabco at p = 0.15 GPa. Experimental diffraction pattern in black, Pawley profile fit in blue and difference curve in grey.

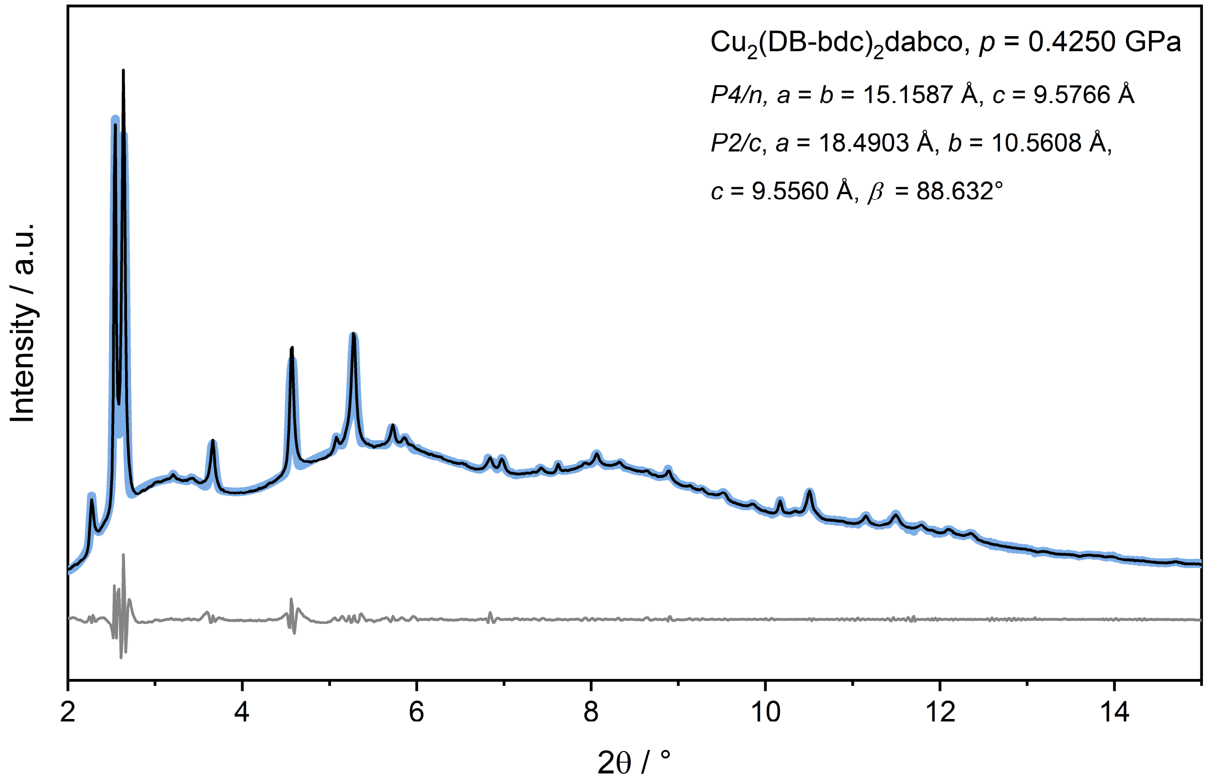

Figure S8. Pawley profile fit $Cu_2(DB\text{-}bcd)_2$dabco at p = 0.425 GPa. Experimental diffraction pattern in black, Pawley profile fit in blue and difference curve in grey.

Table S1. Overview of the cell parameters for Cu_2bdc_2dabco obtained from Pawley profile fits.

p / GPa	*a* / Å	*σ(a)* / Å	*c* / Å	*σ(c)* / Å	*V* / Å^3	*σ(V)* / Å^3	fwhm	R_{wp}	R_{exp}	gof
0.0001	10.835702	0.000212	9.651148	0.000282	1133.165	0.055	0.0246	1.120	4.316	0.260
0.025	10.829627	0.000141	9.642609	0.000287	1130.893	0.045	0.0224	1.444	4.343	0.332
0.050	10.825285	0.000146	9.636583	0.00032	1129.275	0.048	0.0209	1.470	4.380	0.336
0.075	10.821578	0.000143	9.628847	0.000327	1127.597	0.048	0.0203	1.439	4.358	0.330
0.100	10.814708	0.000206	9.624217	0.000393	1125.628	0.063	0.0207	2.063	4.363	0.473
0.125	10.811528	0.000172	9.605719	0.000417	1122.804	0.06	0.0197	1.848	4.384	0.422
0.150	10.8073	0.00021	9.600096	0.000492	1121.269	0.072	0.0212	2.275	4.377	0.520
0.175	10.80113	0.00017	9.594109	0.000376	1119.291	0.056	0.0194	1.812	4.374	0.414
0.200	10.793154	0.000214	9.584057	0.000485	1116.468	0.072	0.0230	1.723	4.407	0.391
0.225	10.782475	0.000248	9.576373	0.000547	1113.366	0.082	0.0262	1.390	4.462	0.312
0.250	10.760952	0.000258	9.569135	0.000634	1108.088	0.091	0.0282	1.094	4.406	0.248
0.275	10.705459	0.000797	9.542009	0.001718	1093.58	0.256	0.0495	0.989	4.491	0.220
0.300	10.705735	0.000794	9.540589	0.001751	1093.473	0.258	0.0508	0.979	4.507	0.217
0.350	10.670831	0.001078	9.514711	0.002739	1083.408	0.381	0.0709	0.756	4.492	0.168
0.400	10.652264	0.001158	9.486915	0.003817	1076.487	0.492	0.0811	0.652	4.435	0.147
0.0001	10.784705	0.001457	9.600742	0.004322	1116.661	0.586	0.0698	0.971	4.730	0.205

Table S2. Overview of the cell parameters for $Cu_2(DB\text{-}bdc)_2dabco$ (space group *P4/n*) obtained from Pawley profile fits between *p* = ambient – 0.425 GPa. Note that from *p* = 0.15 GPa on the data were fitted with a biphasic fit of *P4/n* and *P2/c*. Note that this space group embeds features of two pores per unit cell. Thus, to compare the volumes reported here with the volumes mentioned in the main text, these have to be divided by a factor of two.

p / GPa	*a* / Å	*σ(a)* / Å	*c* / Å	*σ(c)* / Å	*V* / Å^3	*σ(V)* / Å^3	fwhm	R_{wp}	R_{exp}	gof
0.0001	15.261849	0.000451	9.608981	0.000412	2238.163	0.163	0.0227	1.776	3.624	0.490
0.025	15.254496	0.000318	9.605505	0.000349	2235.198	0.123	0.0248	1.957	3.642	0.537
0.050	15.245674	0.000346	9.597336	0.000387	2230.714	0.135	0.0254	2.048	3.629	0.564
0.075	15.234661	0.000408	9.591072	0.000432	2226.039	0.156	0.0275	2.205	3.607	0.611
0.100	15.226999	0.000395	9.582767	0.000451	2221.875	0.156	0.0272	2.170	3.666	0.592
0.125	15.221208	0.000391	9.575937	0.000412	2218.603	0.149	0.0259	2.202	3.664	0.601
0.150	15.219512	0.000555	9.570779	0.00073	2216.913	0.234	0.0239	1.386	1.757	0.789
0.175	15.214983	0.000806	9.568583	0.001098	2215.086	0.346	0.0231	2.093	1.842	1.136
0.200	15.210412	0.000659	9.56801	0.000878	2213.623	0.279	0.0238	1.585	1.892	0.838
0.225	15.20372	0.000922	9.569655	0.001241	2212.055	0.393	0.0242	1.695	1.809	0.937
0.250	15.197282	0.001212	9.583742	0.001036	2213.436	0.427	0.0256	1.442	1.805	0.799
0.275	15.195424	0.001538	9.588381	0.001052	2213.966	0.51	0.0277	1.406	1.856	0.757
0.300	15.186234	0.002344	9.587373	0.001213	2211.057	0.738	0.0280	1.705	1.822	0.936
0.325	15.179758	0.003298	9.585182	0.001358	2208.666	1.009	0.0292	1.867	1.819	1.027
0.350	15.175899	0.004932	9.583431	0.001366	2207.14	1.469	0.0301	1.937	1.841	1.052
0.375	15.164495	0.006305	9.580525	0.001516	2203.156	1.865	0.0309	2.127	1.839	1.157
0.400	15.173683	0.008653	9.578517	0.001533	2205.364	2.54	0.0289	2.150	1.832	1.173
0.425	15.158723	0.009324	9.576608	0.001527	2200.579	2.73	0.0288	2.173	1.852	1.173
0.0001	15.261548	0.000378	9.614548	0.000376	2239.371	0.141	0.0228	2.313	3.719	0.622

Table S3. Overview of the cell parameters for $Cu_2(DB\text{-}bdc)_2dabco$ (space group *P2/c*) obtained from Pawley profile fits between *p* = 0.15 – 0.425 GPa. Note that Rwp, r_ex and gof are the same as in Table S2 because the data were fitted with a biphasic fit from *p* = 0.15 – 0.425 GPa. Note that this space group embeds features of two pores per unit cell. Thus, to compare the volumes reported here with the volumes mentioned in the main text, these have to be divided by a factor of two.

p / GPa	*a* / Å	*σ(a)* / Å	*b* / Å	*σ(b)* / Å	*c* / Å	*σ(c)* / Å	*β* / °	*σ(β)* / °	*V* / Å^3	*σ(V)* / Å^3	fwhm
0.150	18.763936	0.1226	10.387401	0.060822	9.623709	0.319051	89.087	5.543	1875.505	64.383	0.1698
0.175	18.745519	0.071399	10.398804	0.073113	9.631587	0.078622	89.120	3.346	1877.273	21.517	0.1113
0.200	18.826055	0.043752	10.435589	0.052186	9.633629	0.193946	89.063	1.930	1892.379	39.515	0.1416
0.225	18.779686	0.02552	10.409216	0.047864	9.62035	0.07293	89.131	1.647	1880.387	16.887	0.1204
0.250	18.699082	0.008006	10.45977	0.023087	9.621724	0.011479	89.168	0.454	1881.696	4.794	0.0795
0.275	18.653663	0.006593	10.522471	0.018912	9.634333	0.01432	88.750	0.238	1890.602	4.463	0.0700
0.300	18.611351	0.007734	10.510447	0.022292	9.628811	0.021749	88.824	0.266	1883.13	5.89	0.0673
0.325	18.581221	0.007294	10.489205	0.021439	9.607761	0.017824	88.825	0.253	1872.181	5.223	0.0614
0.350	18.553385	0.008795	10.519119	0.025921	9.591369	0.020814	88.615	0.295	1871.355	6.213	0.0601
0.375	18.528971	0.0109	10.532757	0.034855	9.594852	0.022576	88.695	0.273	1872.057	7.683	0.0581
0.400	18.513156	0.011232	10.544251	0.026525	9.564226	0.02672	88.387	0.272	1866.268	7.111	0.0538
0.425	18.49029	0.019469	10.560796	0.030979	9.556012	0.037325	88.632	0.392	1865.492	9.327	0.0547

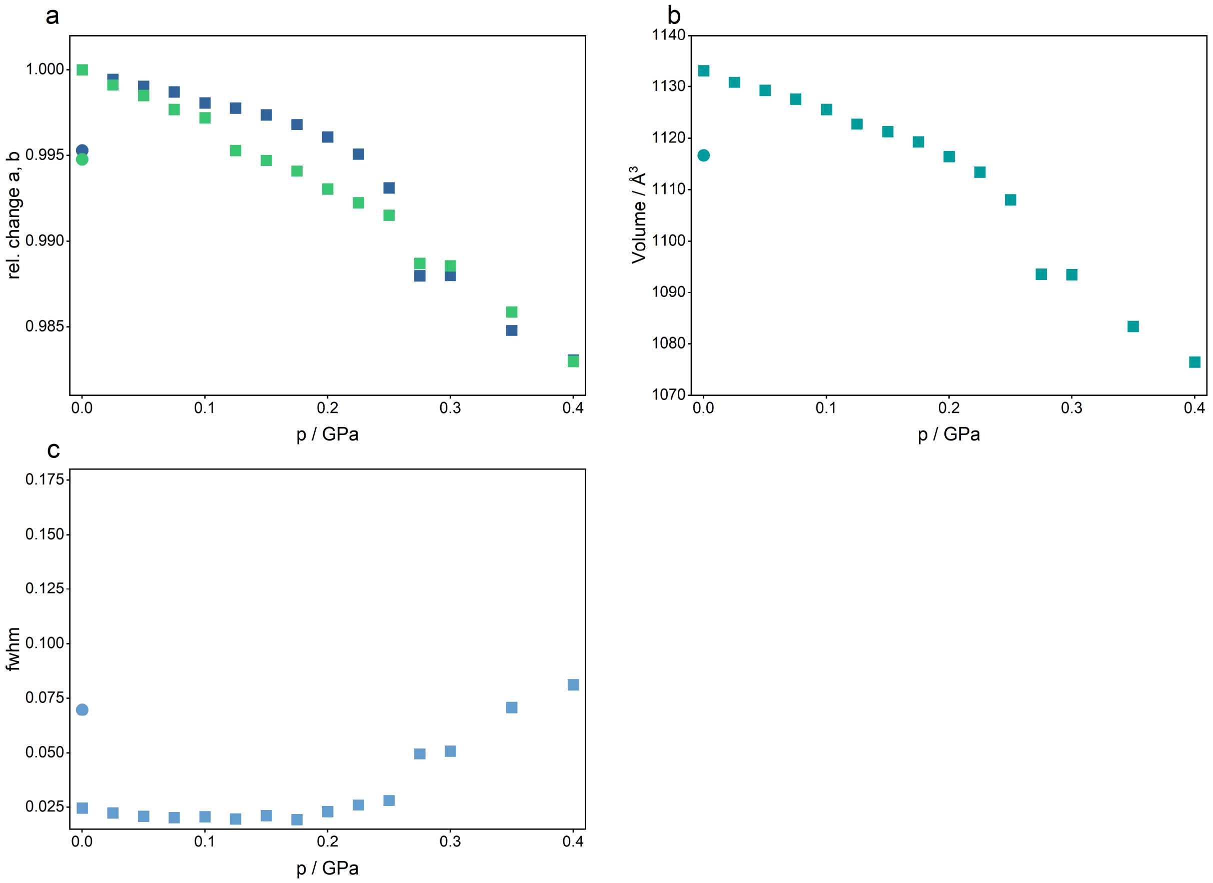

Figure S9. The evolution of **a** lattice parameters (colour code: blue= *a*, green = *c*), **b** volume and **c** fwhm for Cu_2bdc_2dabco as a function of pressure as extracted from Pawley profile fits. Circles indicate the parameter at ambient pressure after the pressure was released.

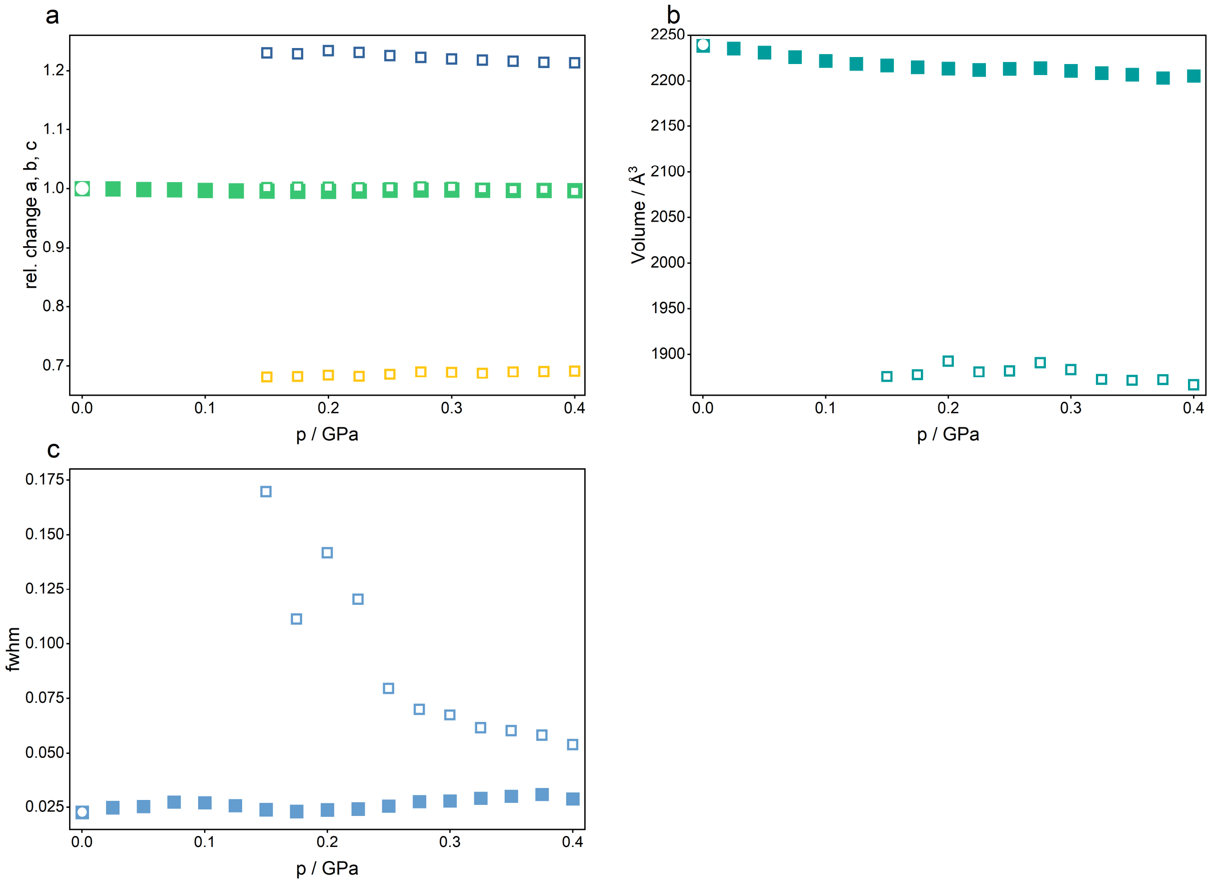

Figure S10. The evolution of **a** lattice parameters (colour code: blue = *a*, yellow = *b*, green = *c*), **b** volume and **c** fwhm for $Cu_2(DB\text{-}bdc)_2dabco$ as a function of pressure as extracted from Pawley profile fits. Filled squares represent the data for the lp phase (*P4/n*) and empty squares for the **np** phase (*P2/n*). Note, that empty circles indicate the parameter at ambient pressure after the pressure was released and are overlapping with the first measurement at ambient pressure. Same applies for lattice parameter c where the data from both phases overlap.

Bulk Modulus

The bulk moduli were derived by fitting a 2nd order Birch-Murnaghan equation of state (BM EoS) to the extracted data. Standard deviations (σ) of lattice parameters and volumes were obtained during the Pawley profile fits and were included in the fitting process of the *V(p)* data with the software EoSFIT-7c.[4] The obtained bulk moduli are K(Cu_2bdc_2dabco) = 14.03 ± 0.20 GPa and K($Cu_2(DB\text{-}bdc)_2dabco$) = 13.46 ± 0.22 GPa. For both materials the datapoints were used before the amorphisation or phase transition occurs: p(Cu_2bdc_2dabco) = ambient – 0.175 GPa and p($Cu_2(DB\text{-}bdc)_2dabco$) = ambient – 0.125 GPa.

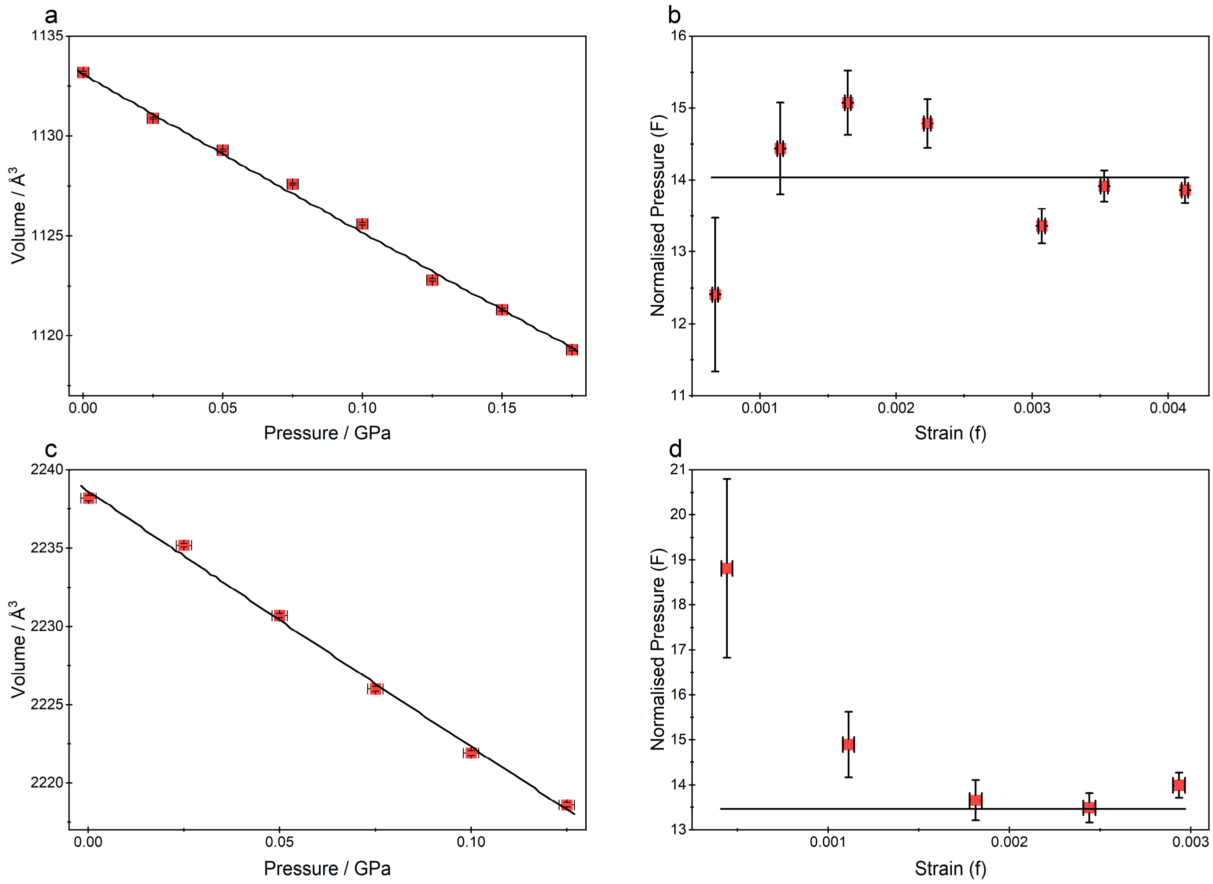

Figure S11. *V(p)* data and EoS fit for Cu_2bdc_2dabco (**a**, **b**) and $Cu_2(DB\text{-}bdc)_2dabco$ (**c**, **d**) shown as *Vp*-plot (**a**, **c**) and *Ff*-plot (**b**, **d**).

Bulk Moduli of Various MOFs

Table S4. List of bulk moduli of various MOFs derived by experiment or simulation.

Material	*K* (GPa)	Ref.
$Cu_2(bdc)_2dabco$	14.0	This work[a]
$Cu_2(DB\text{-}bdc)_2dabco$	13.5	This work[a]
$Cu_2(bdc)_2dabco$	11.3	This work[b]
$Cu_2(DB\text{-}bdc)_2dabco$	10.2	This work[b]
$Cu_2(bdc)_2dabco$	16-18	[5]
$Zn_2(bdc)_2dabco$	13-16	[5]
UiO-66	12-26	[9]
UiO-66	17-22	[10,11]
UiO-66	38	[12]
UiO-67	13	[10]
MOF-5	16-18	[13–16]
HKUST-1	30-35	[17,18]
DUT-52	17	[12]
PCN-57	4.6	[12]
NU-901	7.2	[12]
ZIF-8	6.5	[19]
ZIF-4(Zn)	2-4.4	[20]

[a] Bulk moduli from experimental data in this work; [b] Bulk moduli derived from simulations in this work.

NiDMG as Reference Material

To verify the setup and the pressure applied, Nickel dimethylglyoxime (NiDMG) was used as a reference material because of its known pressure behaviour (see Refs. [21,22]). The capillary was prepared in the same way as the MOFs except that is was not done in the glovebox. 22 HPPXRD patterns were collected between p = ambient and 0.40 GPa including one after releasing the pressure. The step size was Δp = 0.02 GPa.
NiDMG was successfully indexed in the space group *Ibam* (No. 72) and with the cell parameters a = 16.58, b = 10.44, c = 6.46 Å and V = 1120.43 Å^3 as reported by Refs. [21,23,22]. A complete list with all cell parameters and their standard deviations can be found in Table S5. The bulk modulus was K(NiDMG) = 8.31 ± 0.08 GPa which is in accordance with the bulk modulus reported by Takeda *et al.* (K = 8.00 GPa).[22]

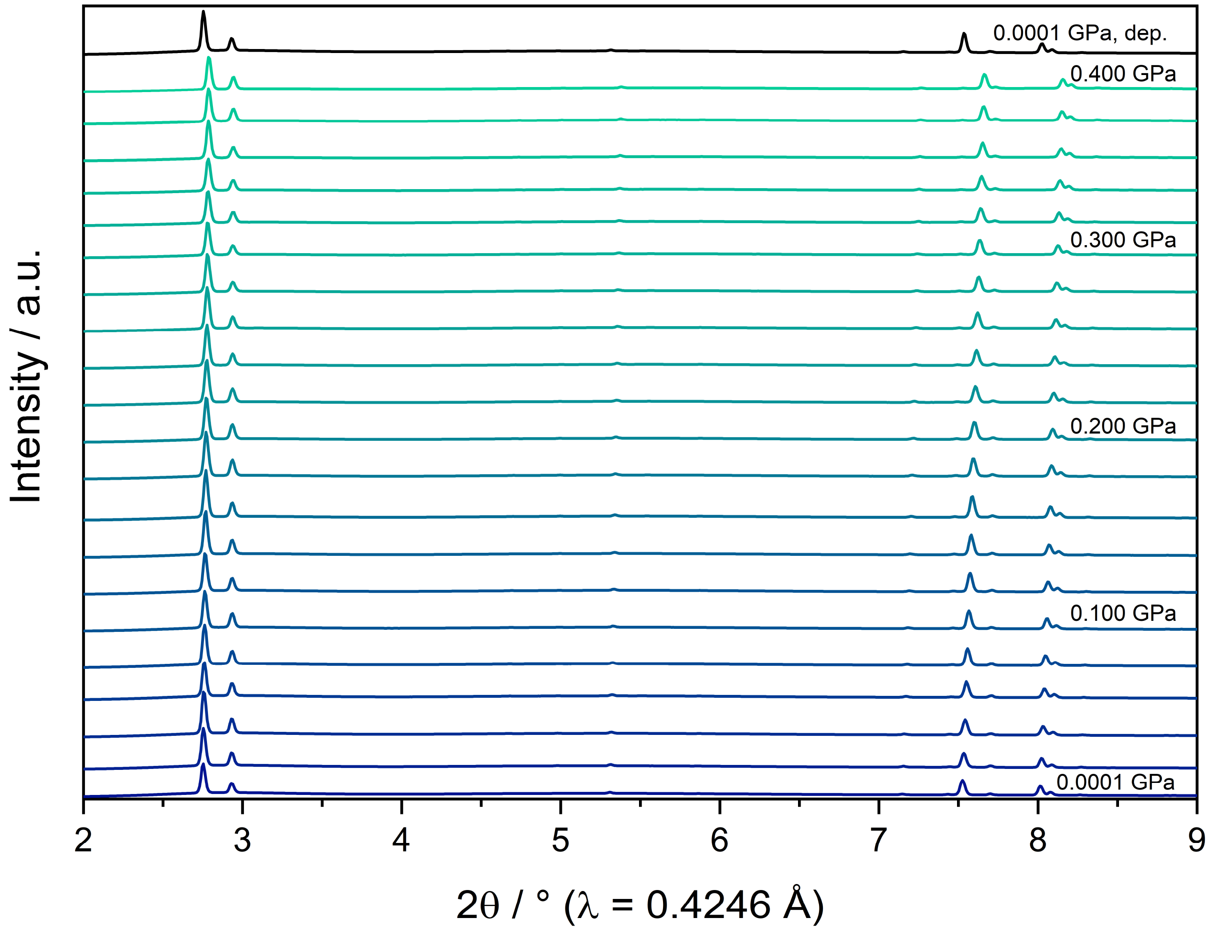

Figure S12. Integrated HPPXRD data of NiDMG taken at p = 0.0001, 0.020, 0.040, 0.060, 0.080, 0.100, 0.120, 0.140, 0.160, 0.180, 0.200, 0.220, 0.240, 0.260, 0.280, 0.300, 0.320, 0.320, 0.340, 0.360, 0.380, 0.400 and 0.0001 GPa after the pressure was released (black).

Table S5. Overview of the cell parameters for NiDMG obtained from Pawley profile fits.

p / GPa	*a* / Å	*σ(a)* / Å	*b* / Å	*σ(b)* / Å	*c* / Å	*σ(c)* / Å	*V* / Å^3	*σ(V)* / Å^3	fwhm	R_{wp}	R_{exp}	gof
0.0001	16.58927	0.00030	10.4414	0.00016	6.46843	0.00004	1120.438	0.027	0.0295	0.857	3.205	0.267
0.020	16.5857	0.00027	10.4292	0.00015	6.46169	0.00004	1117.714	0.025	0.0292	0.957	3.189	0.300
0.040	16.58358	0.00028	10.41642	0.00014	6.45437	0.00004	1114.938	0.025	0.0277	1.036	3.125	0.331
0.060	16.57896	0.00028	10.4052	0.00016	6.44733	0.00004	1112.2111	0.026	0.0276	1.006	3.102	0.324
0.080	16.57652	0.00030	10.3936	0.00015	6.44119	0.00004	1109.752	0.026	0.0284	0.999	3.102	0.322
0.100	16.57455	0.00029	10.383	0.00016	6.43432	0.00004	1107.304	0.026	0.0266	1.036	3.102	0.334
0.120	16.57377	0.00033	10.37511	0.00017	6.42815	0.00004	1105.349	0.029	0.0276	1.135	3.103	0.365
0.140	16.57218	0.00032	10.36249	0.00017	6.42243	0.00004	1102.917	0.028	0.0284	1.195	3.107	0.384
0.160	16.56917	0.00028	10.35457	0.00015	6.41662	0.00003	1100.877	0.025	0.0291	1.062	3.075	0.345
0.180	16.56575	0.00027	10.34539	0.00015	6.41043	0.00004	1098.613	0.024	0.0289	1.061	3.094	0.342
0.200	16.56491	0.00030	10.33661	0.00016	6.40497	0.00004	1096.690	0.027	0.0284	1.140	3.121	0.365
0.220	16.55853	0.00030	10.32904	0.00016	6.39952	0.00004	1094.533	0.026	0.0289	1.096	3.111	0.352
0.240	16.55564	0.00031	10.32115	0.00017	6.39388	0.00004	1092.542	0.028	0.0288	1.049	3.101	0.338
0.260	16.55153	0.00029	10.31236	0.00015	6.3885	0.00004	1090.424	0.026	0.0282	0.990	3.106	0.318
0.280	16.54753	0.00034	10.30376	0.00017	6.38279	0.00004	1088.277	0.029	0.0288	1.048	3.114	0.336
0.300	16.54549	0.00035	10.29905	0.00018	6.37717	0.00004	1086.688	0.030	0.0282	1.059	3.120	0.339
0.320	16.54171	0.00032	10.29046	0.00019	6.37198	0.00005	1084.650	0.030	0.0301	1.054	3.089	0.341
0.34	16.54045	0.00034	10.28341	0.00020	6.36727	0.00005	1083.023	0.031	0.0306	1.079	3.101	0.348
0.360	16.53703	0.00032	10.27566	0.00016	6.36196	0.00004	1081.082	0.027	0.0291	0.995	3.092	0.321
0.380	16.53244	0.00036	10.26952	0.00021	6.35701	0.00005	1079.295	0.033	0.0283	1.207	3.079	0.391
0.400	16.53116	0.00036	10.26381	0.00020	6.35211	0.00005	1077.780	0.032	0.0286	1.166	3.077	0.379
0.0001	16.58840	0.00033	10.42749	0.00016	6.45998	0.00003	1117.418	0.029	0.0275	1.033	3.125	0.330

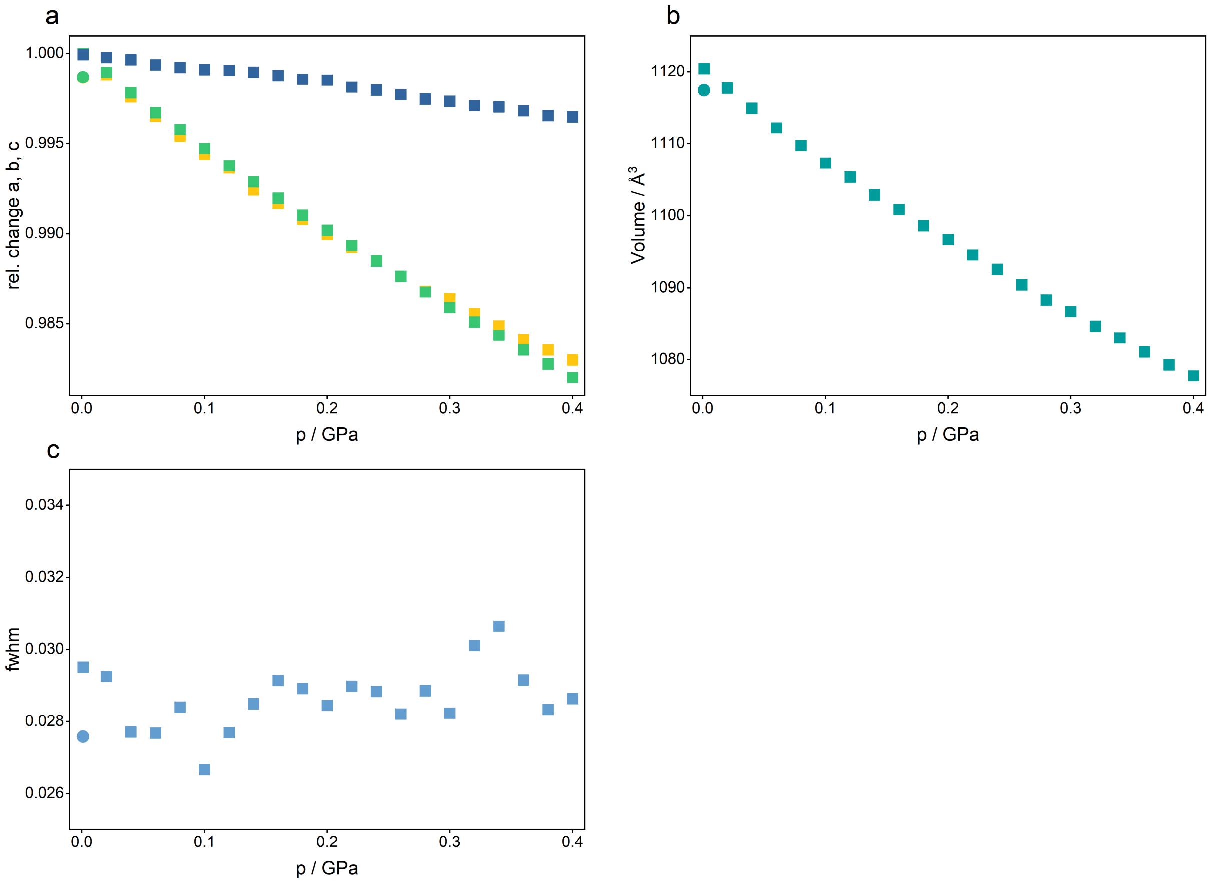

Figure S13. The evolution of **a** lattice parameters (colour code: *a* = blue, *b* = yellow, *c* = green), **b** volume and **c** fwhm for NiDMG as a function of pressure as extracted from Pawley profile fits. Circles indicate the parameter at ambient pressure after the pressure was released.

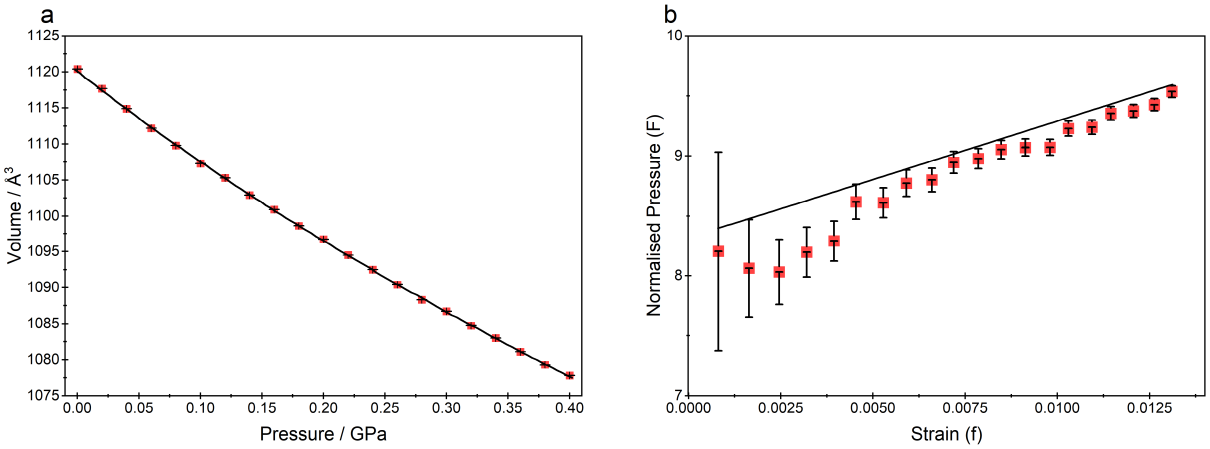

Figure S14. *V(p)* data and EoS fit for NiDMG shown as **a** *Vp*-plot and **b** *Ff*-plot.

Computational Details

The simulation cells used in all simulations contain four formula units and hence four pores of the respective MOFs amounting to 216 atoms for $Cu_2(bdc)_2dabco$ and 424 atoms for $Cu_2(DB\text{-}bdc)_2dabco$. All results shown herein are given per formula unit/pore. Details on how the structures were prepared can be found in the section below. Pressure ramp *NPT* simulations were performed with the LAMMPS implementation of the anisotropic MTTK barostat (from https://github.com/stevenvdb/lammps/tree/newbarostat) using LAMMPS's native pressure ramp functionalities. A thermostat relaxation time of t = 100 fs and a barostat relaxation time of t = 1 ps was used for all runs. For each ps timeframe a structure was written. For the series of $NV(\sigma_a = 0)T$ simulations the average **lp** and **np** structure cell parameters were taken from the pressure ramp runs and the volume was interpolated linearly between these forms using 50 volume windows. For each target volume the structure that differs in volume as little as possible was chosen and scaled to the target volume. The $NV(\sigma_a = 0)T$ simulations were performed with the MTTK barostat using the same settings as above but utilizing a volume constraint. Initial structures were equilibrated for t = 200 ps after which the stress tensor was recorded in t = 1 ns production runs. PXRD patterns were calculated every t = 10 ps using FOX[24] with a Lorentzian line shape width of 0.001 ° and the wavelength of Sn kα at λ = 0.4246 nm also used in the experiment. The *p(V)* equation of state was fitted for $Cu_2(DB\text{-}bdc)_2dabco$ and interpolated using cubic splines for the unfunctionalised MOF. These continuous functions were used for any further analysis of the *p(V)* EoS described below. The bulk modulus *K* was computed from the EoS at the roots of *p(V)* where the slope is negative by numerically estimating the derivative $(\partial P/\partial V)$ from the *p(V)* fit and applying the formula

$$K(V) = -V\left(\frac{\partial P}{\partial V}\right)$$

The Helmholtz free energy A was obtained via numerically integrating the *p(V)* EoS starting from the root of *p(V)* corresponding to the large pore by using

$$\Delta A(V) = A(\mathrm{V}) - A(V_{ref}) = -\int_{V_{ref}}^{V} P(V')dV'$$

From the average of the internal Energy *U(V)* of each simulation, the entropic energy contribution *TS(V)* and the entropy *S(V)* was computed via the fundamental relation *U* = *A*-*TS*. All thermodynamic quantities were arbitrarily shifted so the minimum corresponds to the origin, since their absolute differences do not carry any meaning without having sampled a proper path that connects them.
The vibrational entropy contributions in the harmonic approximation were computed as follows: After a tight optimization of the initial structural models, the Hessian matrix of second derivatives was computed by finite differencing of the forces using MOF-FF in LAMMPS. Imaginary frequencies were assured to be absent. The entropy at T = 300 K was computed from the Hessian using phonopy[25] v2.1.4. using a 12x12x12 q-point mesh, which we assured to be reasonably well converged. The calculations for $Cu_2(DB\text{-}bdc)_2dabco$ were performed for a single snapshot for each Group-1 structure at a volume of V = 937.5 Å^3 (**np**) and V = 1125 Å^3 (**lp**). The averaged entropy penalty term $-T\Delta S$ obtained in this way amounts to 21.1 kJ·mol^{-1} with a standard deviation of 2.9 kJ·mol^{-1}.
All simulation data are available upon reasonable request. Run scripts and the analysis routines are accessible at the CMC groups software repository at https://github.com/cmc-rub/supporting_data/tree/master/XX-Keupp-XX_2020

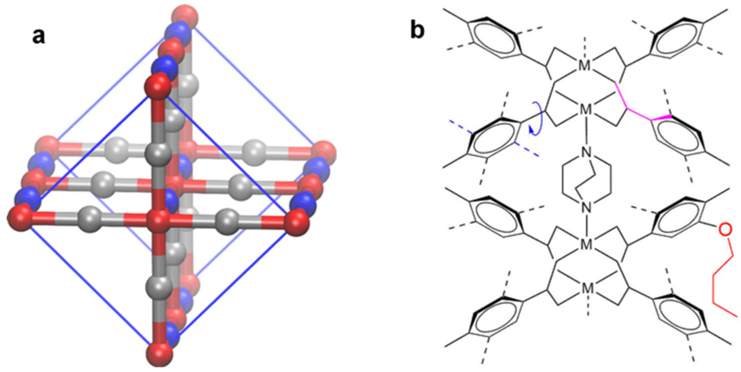

Figure S15. a Blueprint of the simulation box. Colour code: red = paddlewheel unit; blue = dabco; grey = fu-bdc linker. The simulation box consists of two layers with two Paddlewheels and four linkers, respectively giving rise to a total of four paddlewheel units. **b** Excerpt from the simulated structural model. The carboxylic oxygen atoms forming most of the paddlewheel unit are not shown for clarity. The coloured parts denote the important regions. Blue, magenta: a rotation around this torsion results in a different configuration of the arms attached, as now the 'lower' one points towards the depicted paddlewheel. red: the configuration of the side chains can be different per configuration; however, the conformers are thermally accessible.

Whereas the model preparation of $Cu_2(bdc)_2dabco$ is relatively straight forward, the disorder present in longer side chains poses a problem in the simulation of the free energy profiles for these systems: We have recently pointed out that the simulations of functionalised MOFs as the herein presented $Cu_2(DB\text{-}bdc)_2dabco$ strongly depend on the initial linker configuration, since within the accessible simulation timescales of several nanoseconds a linker flip is observed only very rarely. Therefore, different initial configurations do not transform into each other, but the result we obtain is that of an infinitely large crystallite composed solely of this single configuration. Initial structural models derived from experiment are not readily available as the single crystal data reported by

Henke *et al.*[1,26] cannot uniquely be transformed into an initial model to use for the simulations because the side chains are not resolved (even at T = 87 K) and the positions of the oxo-moieties are disordered. Thus, there are many different configurations that could be valid structures based on the comparison to the XRD data.
We therefore constructed many trial initial structural models from the blueprint shown in Figure S15 using the weaver code[27] followed by a lattice optimization step: 32 individual starting structures with random phenyl orientations (*i.e.* random torsion angles as indicated by the blue and magenta colours in Figure S15) were generated, optimized and used to run the NPTs from which the p(V) EoS is computed.

The evaluation of the trial structures of $Cu_2(DB\text{-}bdc)_2dabco$ is done by comparing PXRD patterns computed along and averaged over the $NV(\sigma_a = 0)T$ trajectories at a cell volume of V = 937.5 Å^3. Figure S16 shows all 32 PXRDs compared to the experimental high-pressure pattern at p = 0.4 GPa.

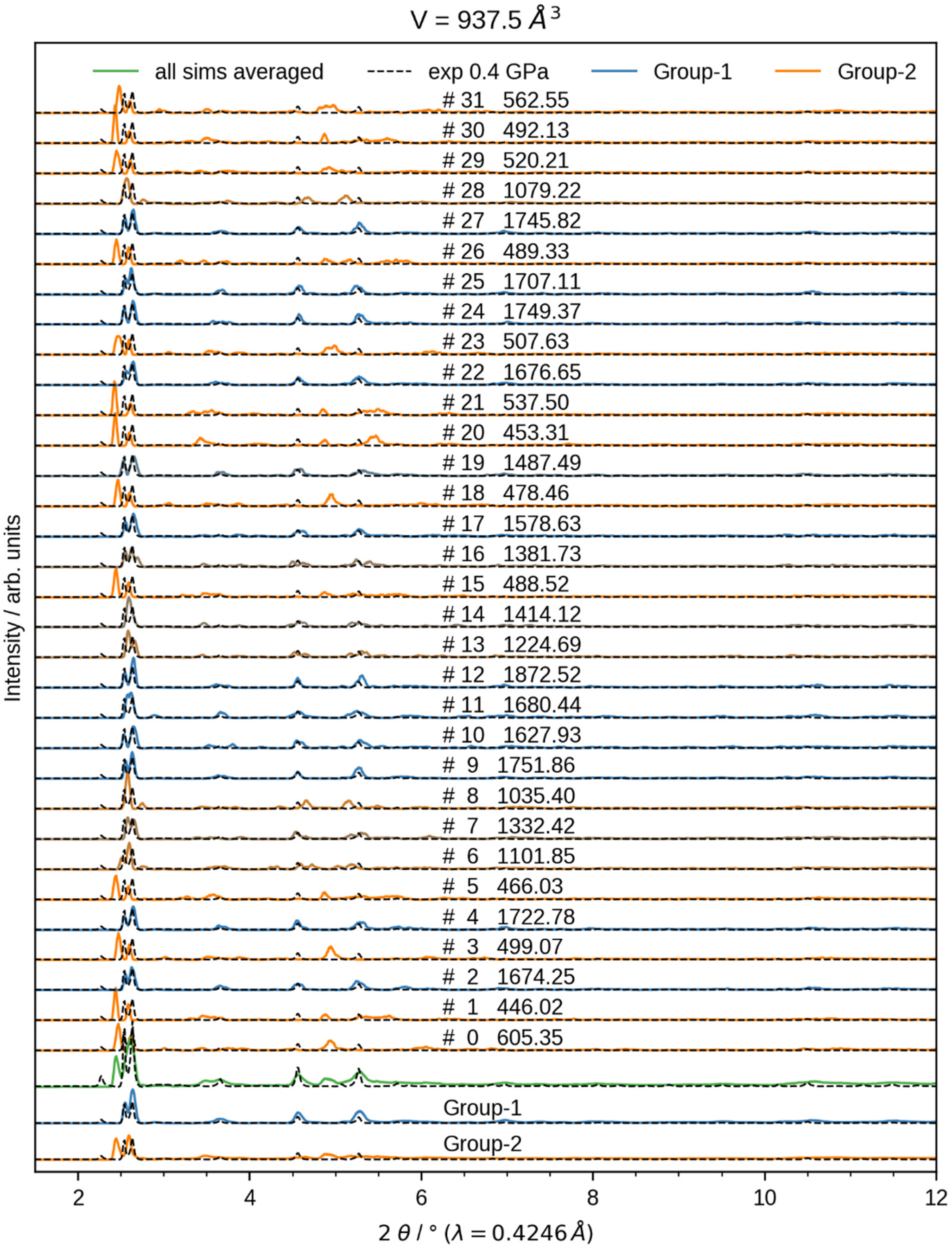

Figure S16. An overview of all simulated PXRD patterns at V = 937.5 Å^3 compared to the experimental high-pressure PXRD pattern at p = 0.4 GPa. The text next to each plot denotes the structure index and the calculated similarity between the experimental and the simulated pattern according to the Rietveld fit inspired value function.

To assess the similarity between two PXRD patterns, we used the Rietveld fit inspired value function:

$$f = \int_{\theta_{min}}^{\theta_{max}} I_{obs}\, I_{calc} d\theta \approx \sum_{i=0}^{n} I_{obs}(\theta_i) I_{calc}(\theta_i)\, \Delta\theta$$

estimated by means of numerical integration on a grid of $\Delta\theta$ = 0.01°. These are the values listed in Figure S16. A similarity above f = 1500 is considered a full match and a value below f = 1000 is considered a full mismatch. In between we linearly interpolated the two colours, but for a zero-one decision, we used a threshold of f = 1300. The averages of Group-1 and Group-2 are thus calculated from all structures featuring an f smaller or larger than 1300, respectively. The averaged PXRD patterns are shown in Figure 4 in the main text. Interestingly, after this classification into Group-1 and Group-2, where Group-1 are the structures with matching PXRD patterns and Group-2 are those that do not match, their distinct behaviour can also be observed in the PXRD patterns along the pressure ramp trajectories: Whereas the Group-1 structures show a step as observed in the experiment, the Group-2 structures do not. Instead one can follow their PXRD pattern shifting continuously as the pressure increases.

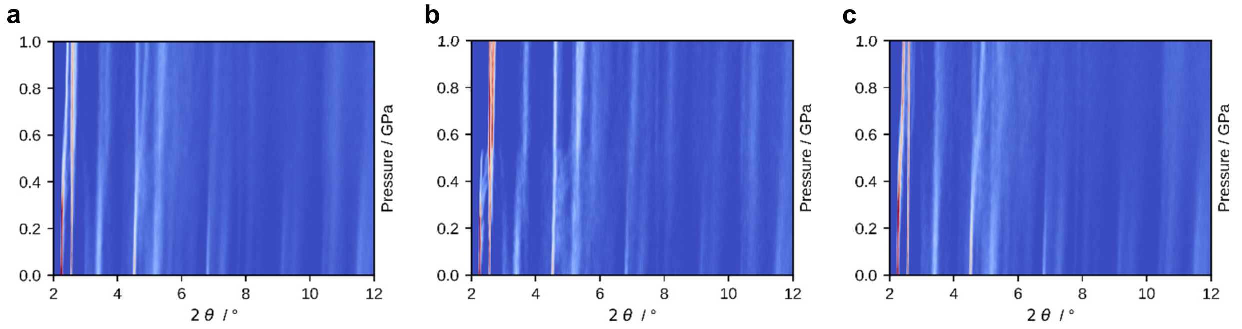

Figure S17. PXRD patterns computed along the pressure ramp *NPT* simulations for Cu_2(DB-bdc)$_2$dabco. **a** averaged over all 32 structures; **b** averaged over the Group-1 structures; **c** averaged over the Group-2 structures.

Cell Parameters

The cell parameters were averaged for each $NV(\sigma_a = 0)T$ simulation. Figure S19 shows the cell lengths and Figure S20 the cell angles, respectively. All Group-1 structures compare very well with the experimental cell angles, whereas in the Group-2 structures qualitative differences are found. In general Group-2 type structures change their aspect ratio to a lesser extent or not at all, whereas all Group-1 structures have a very similar cell angles vs volume profile. Group-1 structures differ in itself in the point at which the cell lengths *a* and *b* start to differ from each other. In any case this point is close to the **lp** cell lengths.

Cell angles as a function of cell volume differ more from the experimental values. In general, the amplitude by which cell angles differ from 90 ° is often higher and even the **lp** form volumes of some of the Group-1 structures adopt cell angles different from 90 °. We attribute the flexibility of the cell angles to the respective linker configurations of the MOF combined with our small simulation cell comprised of only four formula units of the MOF material. Neighbouring pore environments will allow for certain distortions of a given pore and restrict others, an effect we can only capture in larger simulation cells. Probably in experimentally derived cell angles by means of PXRD fits, these effects average out towards a small deviation in the cell angles of only 1.5 °.

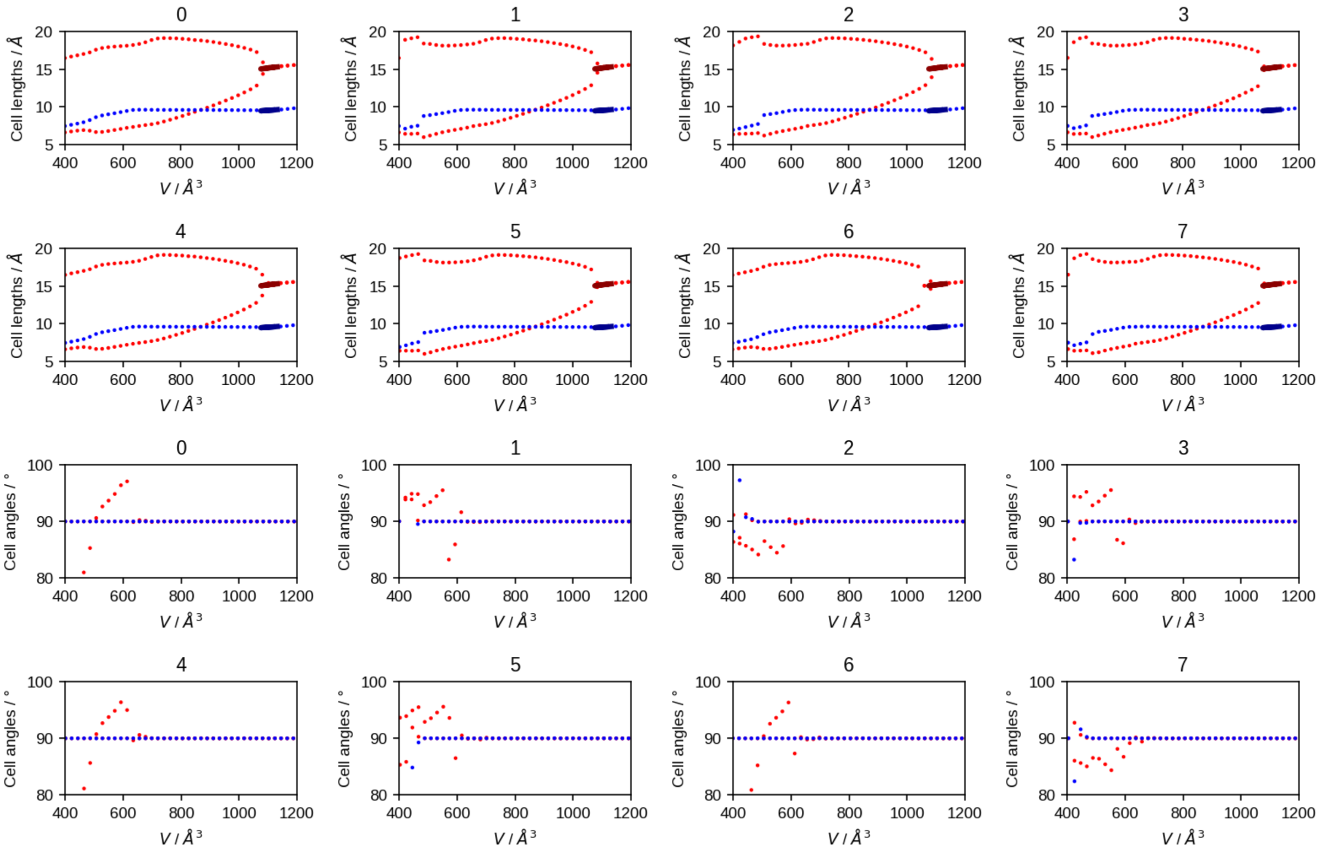

Figure S18. Cell lengths and angles as a function of cell volume of $Cu_2(bdc)_2dabco$. The experimental cell angles are plotted as thick lines. The plot titles show the structure index.

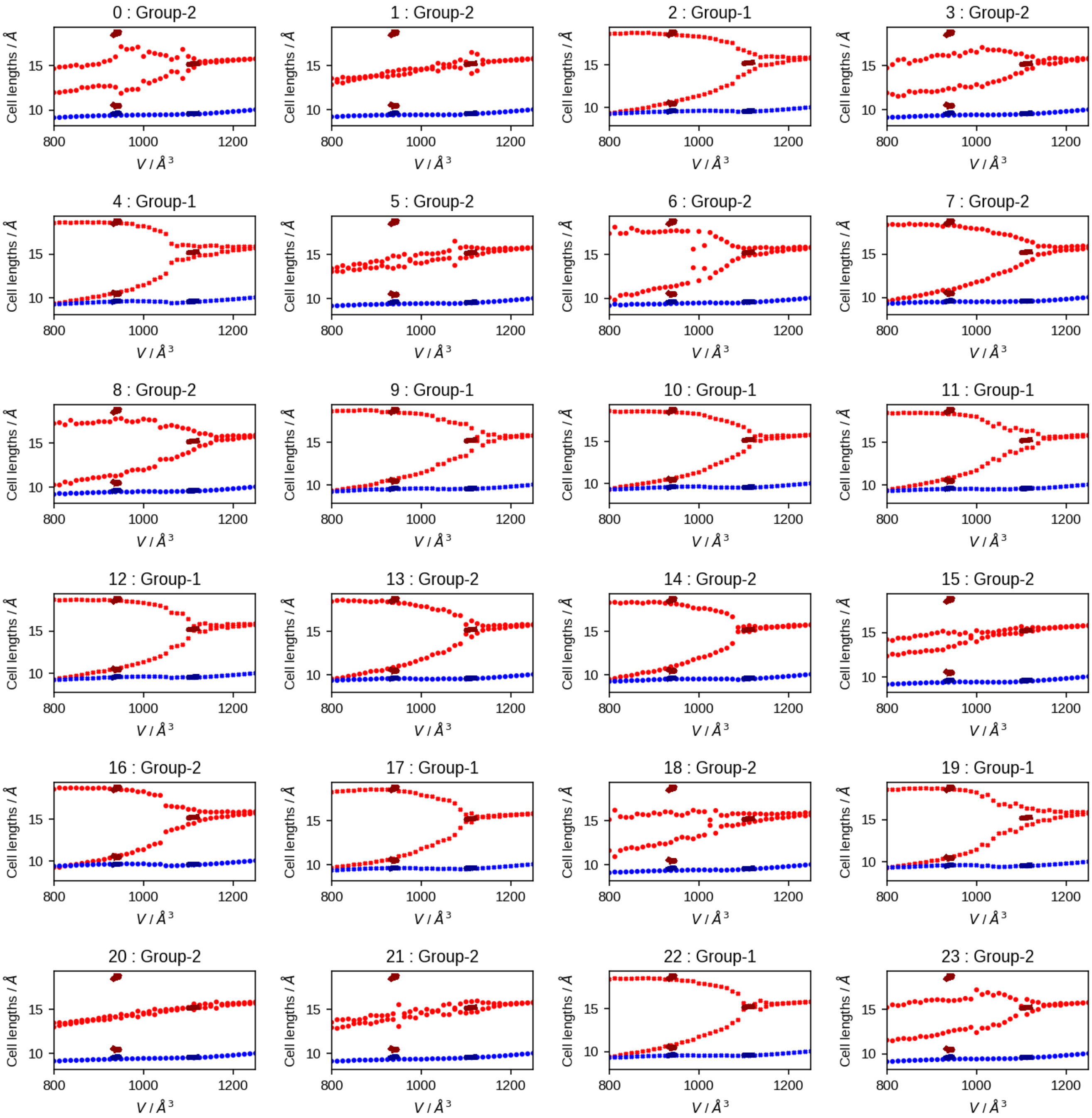

Figure S19. Cell lengths as a function of cell volume of Cu_2(DB-bdc)$_2$dabco . The experimental cell lengths are plotted as thick lines. The plot titles show the structure index and the group to which it belongs.

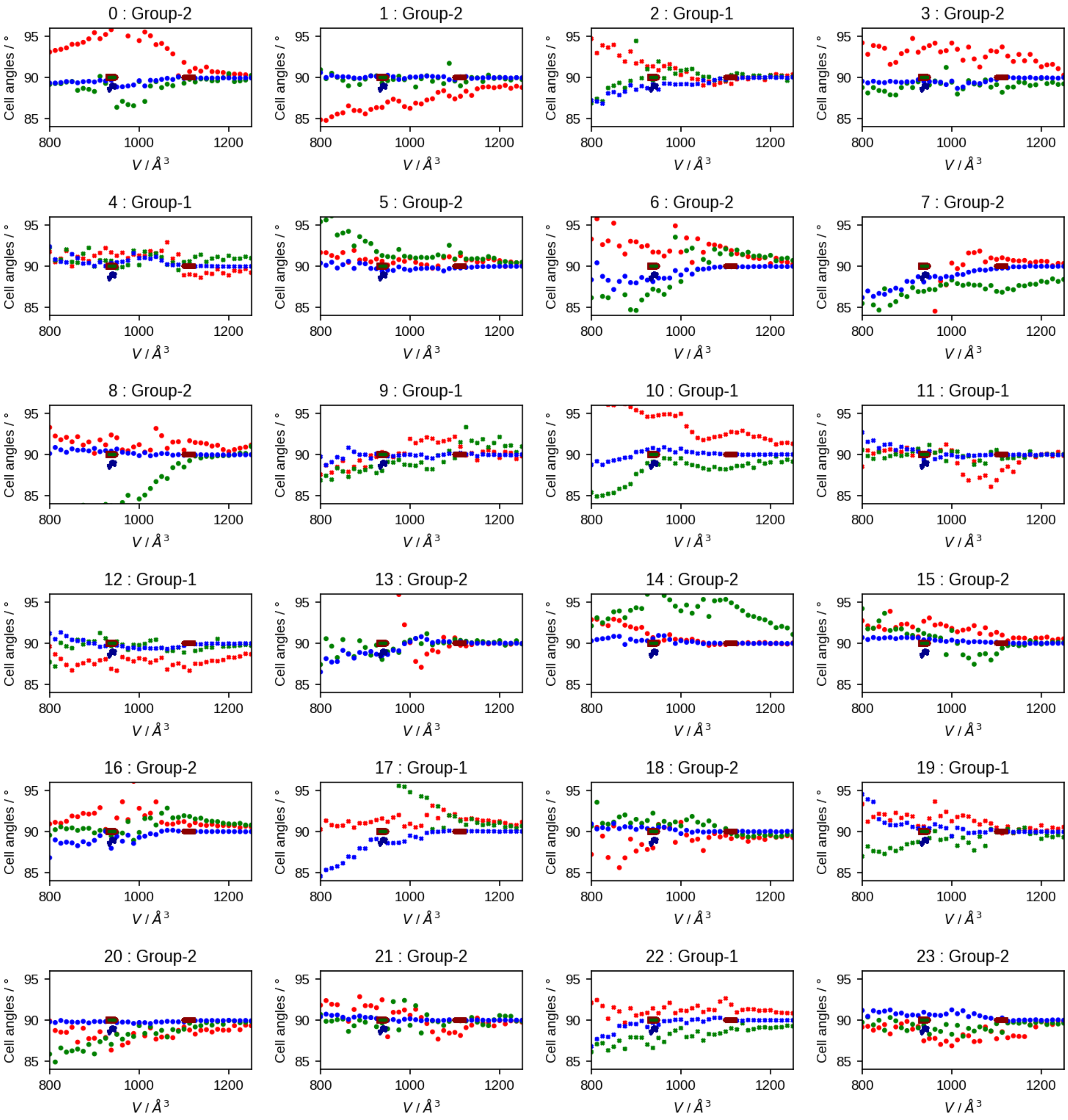

Figure S20. Cell angles as a function of cell volume of Cu_2(DB-bdc)$_2$dabco. The experimental cell angles are plotted as thick lines. The plot titles show the structure index and the group to which it belongs.

Thermodynamics of $Cu_2(DB\text{-}bdc)_2dabco$: Internal Energy, Free Energy and Entropy

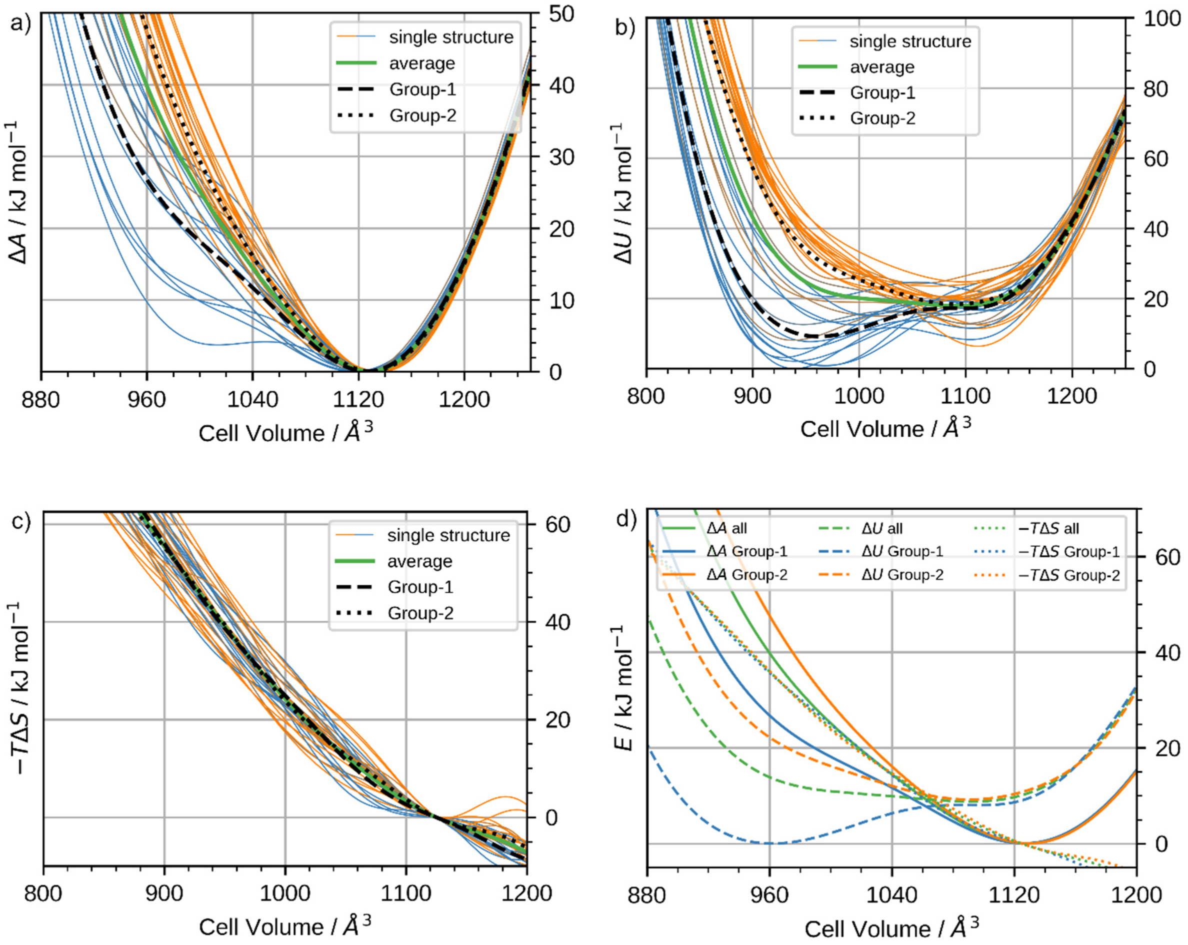

Figure S21. a Helmholtz free energy, **b** internal energy and **c** entropy contribution for the 32 $Cu_2(DB\text{-}bdc)_2dabco$ structures as a function of the cell volume V. The colour code for **a**, **b** and **c** was taken from the PXRD match described above. The blue curves are those structures that match the PXRD. Non-matching structures are shown in orange and the average of all is shown in green. The thick lines in **a** – **c** are the averages which are collected and shown all in one plot in **d**. Note that one single structure has a shallow free energy minimum at a volume corresponding to the **np** phase, thus rendering it a metastable state.

From the *p(V)* EoS (*cf.* Figure 3) ΔA was computed by numerical integration, and from the average of the total energy, the internal Energy ΔU was obtained. Since the free energy difference of two different structures cannot be inferred in this way, each individual structure was shifted to its own origin. For ΔU it is different, since the very same force field terms give rise to a comparable total energy. Therefore, all structures were shifted to the global minimum of the global minimum structure. The plots above show the data derived from polynomial fits of *p(V)* of the raw data points every V = 12.5 Å^3. Experimentally it is observed that $Cu_2(DB\text{-}bdc)_2dabco$ at ambient conditions remains at its **lp** form even after removal of solvent or guest molecules. This is properly predicted by the global free energy minimum of all structures at V = 1125 Å^3. As seen from the *p(V)* plots, a volume step at elevated pressures between p = 0.1 and 0.3 GPa is present for some of the simulated structures, but none of them is a metastable phase at ambient pressure. This is also in accordance with the fact, that in experiment after compression, the phase transition is entirely reversible. Interestingly, the internal energy of the Group-1 structure models has its global minimum at a volume corresponding to a **np** form (approx. V = 950 Å^3). It is the entropy that adds an average of 42 kJ mol^{-1} per simulation cell to the free energy to render the **np** form a high-pressure phase that cannot be trapped in a metastable state at ambient temperatures. Also note that the internal energies of the **lp** form are almost indistinguishable when comparing Group-1 and Group-2, which may hint towards a strong disorder in this system, since $Cu_2(DB\text{-}bdc)_2dabco$ is synthesized in the **lp** form at elevated temperature.

Computing the Work Released during the Phase Transitions

The work released during the phase transitions are calculated according to the formula $W = -p\Delta V$, where ΔV is the volume difference between **lp** and **np** forms and p is the pressure at which the phase transition occurs. In order to relate this quantity to an increase in temperature under the assumption that no energy is dissipated elsewhere, we used the formula

$$\frac{E_{kin}}{3N} = \frac{k_B T}{2}$$

and use the work as kinetic energy. N is the number of degrees of freedom, in this case three times the number of atoms in one formula unit minus three (to account for translational invariance.). For $Cu_2(bdc)_2$dabco we have utilized p = 0.195 GPa and dV = 525 $Å^3$, which corresponds to a work of W = 61.6 kJ·mol^{-1} and an increase in temperature of about ΔT = 92 K. For Cu_2(DB-bdc)$_2$dabco we used p = 0.29 GPa and dV = 187.5 $Å^3$, which amounts to a work W = 32.7 kJ·mol^{-1} and an increase in temperature of about ΔT = 25 K.

Structural Details via Collective Variables

In order to analyse the individual structures on an atomistic level, a selection of collective variables shown in Figure S22 was computed along all trajectories of all $NV(\sigma_a = 0)T$ simulations.

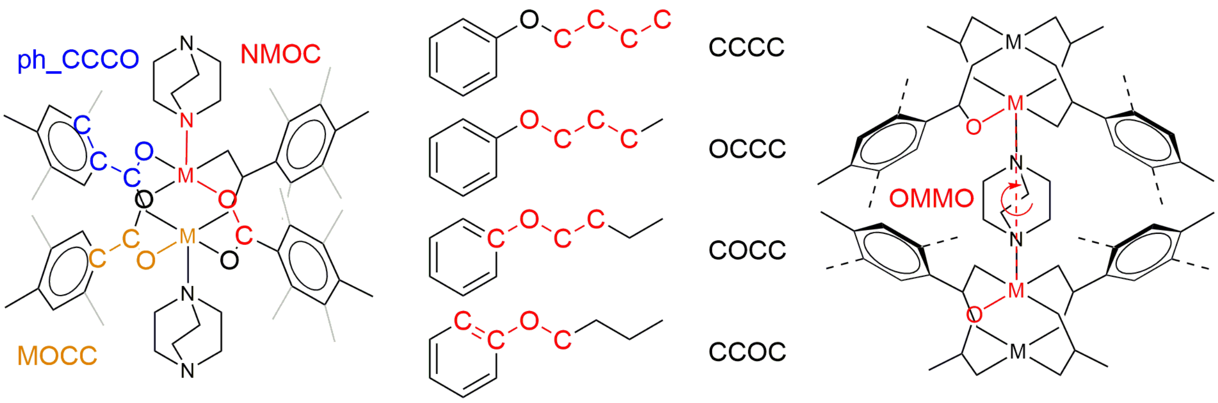

Figure S22. Selected collective variables to analyse the atomistic structures of the simulations.

The quest is to find those collective variables, where the two sets of structures that do (Group-1) and do not (Group-2) fit the **np** PXRD pattern differ. The histograms of the collective variables shown in Figure S23 were collected along the $NV(\sigma_a = 0)T$ trajectories of the given volume. The distributions of the **lp** form (at $V = 1125$ Å^3) collective variables are almost identical, with only slight variations in the COCC, OCCC and CCCC profiles that point towards the side chains being a little more elongated and stiff in case of the Group-1 structures. For the **np** forms this trend is amplified as the peaks for the Group-1 structures get sharper whereas the peaks for the Group-2 structures get shallower, indicating a slightly larger degree of coiling for the Group-2 structures. In contrast to that, the collective variables OMMO, MOCC and ph_CCCO are very different in the **np** forms comparing the Group-1 and the Group-2 structures with the largest difference residing in the distribution of the OMMO collective variable. The OMMO torsion angle describes a rotation of two paddlewheel units stacked in z-direction in a counter-rotating fashion, leading to the loss of the alignment of the linkers as one looks at the structure along the z-direction. We believe that this effect is what stiffens the structures throughout the simulations. This change is accompanied with a distorted paddlewheel unit as the MOCC torsion shows a larger change and hence a wider distribution for the Group-2 structure in the **np** forms. Finally, for the ph_CCCO torsion there is even a qualitative difference comparing the two sets of structures: In the **np** form, the Group-1 structures show four maxima in the 0 ° region, whereas the Group-2 structures show only two peaks, which are even narrowed down and shifted towards 0 ° during the **lp** to **np** transition. Whereas all other collective variables statistics are very similar in the **lp** phase for the two sets of structures, the ph_CCCO torsion profile is very different even in the **lp** phase, indicating that it is the main reason for the apparent differences in the phase transition behaviours simulated herein. Note that this collective variable was sampled uniformly for each of the eight phenyl moieties of the 32 investigated starting structures in the construction process.

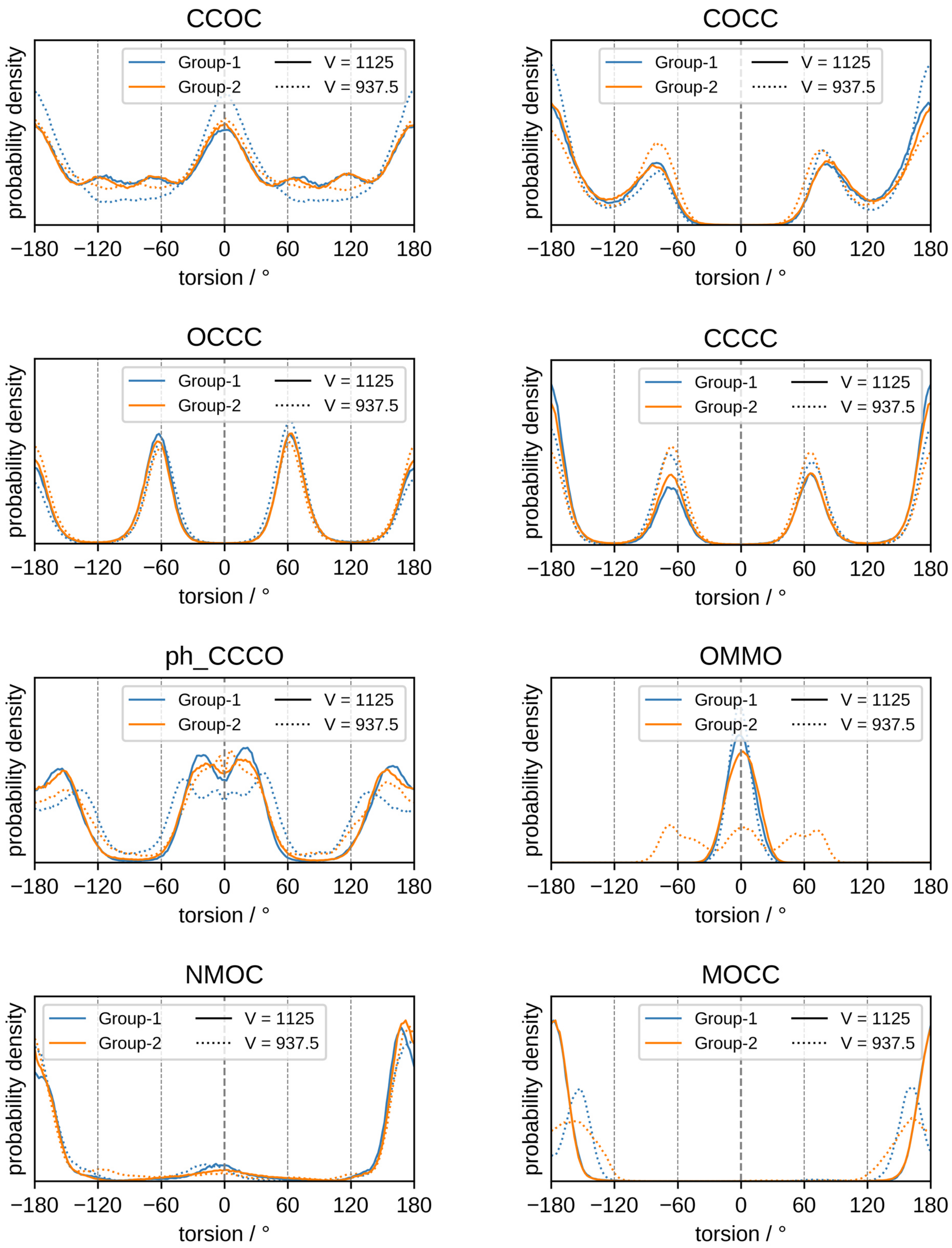

Figure S23. Histograms of collective variables averaged Group-1 structures (blue) and the Group-2 structures (orange). The solid plots denote the **lp** form at V = 1125 Å^3, whereas the dotted plots correspond to the **np** form at V = 937.5 Å^3.

Variability of the Linker Orientations of the $Cu_2(DB\text{-}bdc)_2$dabco Simulations

The entire system is comprised of eight different sites where the functionalized bdc linker is inserted. For each of these different sites, a uniform random orientation angle for the linker is drawn and the linkers is rotated accordingly. The number of distinct configurations depends on the question how these sampled orientations change during the simulation. If we assume that there are four distinct stable orientations, it will amount to 4^8= 65.536 different configurations. The data to analyse this is readily available as the ph_CCCO collective variables were computed for all trajectories. Below is a figure displaying the individual contributions of all 16 torsion profiles that give rise to the total histogram also shown in the Figure S23. The data presented here is at V = 1125 Å^3 for the Group-1 structures of $Cu_2(DB\text{-}bdc)_2$dabco. We observe the general trend that this torsion deviates from the 0 / 180°angle, but it is not a clean cut, but takes into account both this in-plane configuration and the ± 30° deviation from it within a single simulated structure.

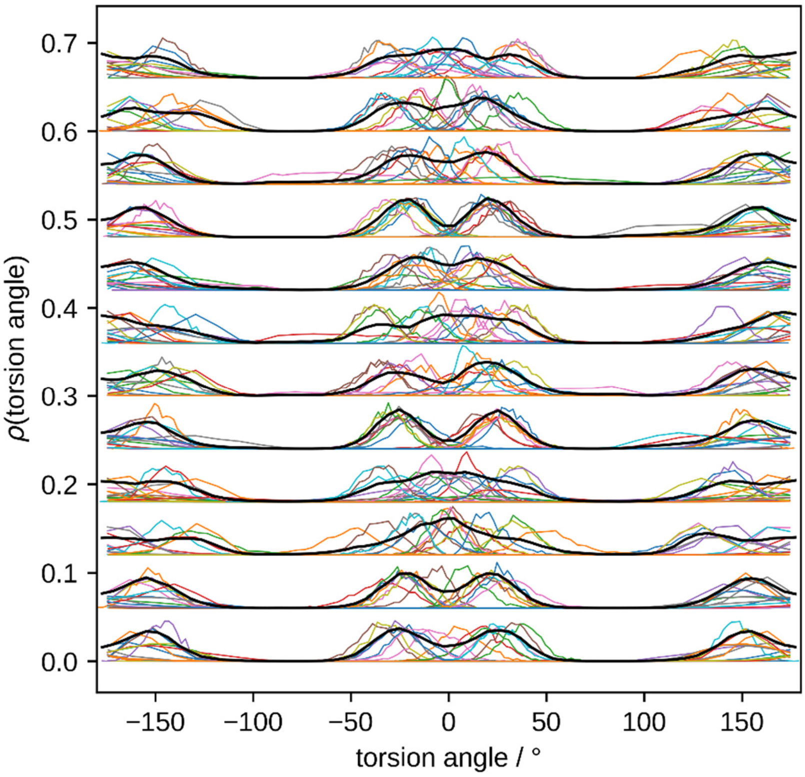

Figure S24. Histograms of the ph_CCCO collective variable for the Group-1 type structure. The coloured lines denote one single collective variable, the black lines correspond to the histogram of all individual collective variables. The latter have been scaled by a factor of five for visibility.

References

[1] S. Henke, A. Schneemann, A. Wütscher, R. A. Fischer, *J. Am. Chem. Soc.* **2012**, *134*, 9464.
[2] N. J. Brooks, Gauthe, Beatrice L. L. E., N. J. Terrill, S. E. Rogers, R. H. Templer, O. Ces, J. M. Seddon, *Rev. Sci. Instrum.* **2010**, *81*, 1.
[3] A. A. Coelho, *J. Appl. Crystallogr.* **2018**, *51*, 210.
[4] R. J. Angel, M. Alvaro, J. Gonzalez-Platas, *Z. Kristallogr. - Cryst. Mater.* **2014**, *229*.
[5] J. Wieme, S. M. J. Rogge, P. G. Yot, L. Vanduyfhuys, S.-K. Lee, J.-S. Chang, M. Waroquier, G. Maurin, V. van Speybroeck, *J. Mater. Chem. A* **2019**, *7*, 22663.
[6] I. Schwedler, S. Henke, M. T. Wharmby, S. R. Bajpe, A. K. Cheetham, R. A. Fischer, *Dalton Trans.* **2016**, *45*, 4230.
[7] T. Hahn (Hrsg.) *International Tables for Crystallography*, *Vol. A*, Springer, Dordrecht, **2002**.
[8] S. Henke, R. Schmid, J.-D. Grunwaldt, R. A. Fischer, *Chem. Eur. J.* **2010**, *16*, 14296.
[9] S. Dissegna, P. Vervoorts, C. L. Hobday, T. Düren, D. Daisenberger, A. J. Smith, R. A. Fischer, G. Kieslich, *J. Am. Chem. Soc.* **2018**, *140*, 11581.
[10] S. M. J. Rogge, J. Wieme, L. Vanduyfhuys, S. Vandenbrande, G. Maurin, T. Verstraelen, M. Waroquier, V. van Speybroeck, *Chem. Mater.* **2016**, *28*, 5721.
[11] P. G. Yot, K. Yang, F. Ragon, V. Dmitriev, T. Devic, P. Horcajada, C. Serre, G. Maurin, *Dalton Trans.* **2016**, *45*, 4283.
[12] L. R. Redfern, L. Robison, M. C. Wasson, S. Goswami, J. Lyu, T. Islamoglu, K. W. Chapman, O. K. Farha, *J. Am. Chem. Soc.* **2019**, *141*, 4365.
[13] A. J. Graham, D. R. Allan, A. Muszkiewicz, C. A. Morrison, S. A. Moggach, *Angew. Chem., Int. Ed.* **2011**, *50*, 11138.
[14] H. Wu, T. Yildirim, W. Zhou, *J. Phys. Chem. Lett.* **2013**, *4*, 925.
[15] A. Samanta, T. Furuta, J. Li, *J. Chem. Phys.* **2006**, *125*, 84714.
[16] M. Mattesini, J. M. Soler, F. Ynduráin, *Phys. Rev. B* **2006**, *73*.
[17] B. Lukose, B. Supronowicz, P. St. Petkov, J. Frenzel, A. B. Kuc, G. Seifert, G. N. Vayssilov, T. Heine, *Phys. Status Solidi B* **2012**, *249*, 335.
[18] K. W. Chapman, G. J. Halder, P. J. Chupas, *J. Am. Chem. Soc.* **2008**, *130*, 10524.
[19] K. W. Chapman, G. J. Halder, P. J. Chupas, *J. Am. Chem. Soc.* **2009**, *131*, 17546.
[20] P. Vervoorts, C. L. Hobday, M. G. Ehrenreich, D. Daisenberger, G. Kieslich, *Z. Anorg. Allg. Chem.* **2019**, *645*, 970.
[21] I. F. Bruce-Smith, B. A. Zakharov, J. Stare, E. V. Boldyreva, C. R. Pulham, *J. Phys. Chem. C* **2014**, *118*, 24705.
[22] K. Takeda, J. Hayashi, I. Shirotani, H. Fukuda, K. Yakushi, *Mol. Cryst. Liq. Cryst.* **2006**, *460*, 131.
[23] D. E. Williams, G. Wohlauer, R. E. Rundle, *J. Am. Chem. Soc.* **1959**, *81*, 755.
[24] V. Favre-Nicolin, R. Cerný, *J. Appl. Crystallogr.* **2002**, *35*, 734.
[25] A. Togo, I. Tanaka, *Scr. Mater.* **2015**, *108*, 1.
[26] S. Henke, A. Schneemann, R. A. Fischer, *Adv. Funct. Mater.* **2013**, *23*, 5990.
[27] J. Keupp, R. Schmid, *Faraday Discuss.* **2018**, *211*, 79.
